# Capturing SNP Association across the NK Receptor and HLA Gene Regions in Multiple Sclerosis by Targeted Penalised Regression Models

**DOI:** 10.3390/genes13010087

**Published:** 2021-12-29

**Authors:** Sean M. Burnard, Rodney A. Lea, Miles Benton, David Eccles, Daniel W. Kennedy, Jeannette Lechner-Scott, Rodney J. Scott

**Affiliations:** 1 School of Biomedical Sciences and Pharmacy, University of Newcastle, Callaghan, NSW 2308, Australia; rodney.a.lea@gmail.com; 2 Centre for Brain and Mental Health (CBMHR), Hunter Medical Research Institute (HMRI), New Lambton Heights, NSW 2305, Australia; jeannette.lechner-scott@health.nsw.gov.au; 3 Centre of Genomics and Personalised Health, School of Biomedical Sciences, Queensland University of Technology, Kelvin Grove, QLD 4059, Australia; 4 Human Genomics, Kenepuru Science Centre, Institute of Environmental Science and Research, Wellington 5240, New Zealand; miles.benton84@gmail.com; 5 Malaghan Institute of Medical Research, Wellington 6242, New Zealand; bioinformatics@gringene.org; 6 Australian Centre of Excellence for Mathematical and Statistical frontiers, Queensland University of technology, Brisbane, QLD 4000, Australia; d.w.kennedy1992@gmail.com; 7 School of Medicine and Public Health, University of Newcastle, Callaghan, NSW 2308, Australia; 8 Department of Neurology, John Hunter Hospital, New Lambton Heights, NSW 2305, Australia; 9 Division of Molecular Medicine, NSW Health Pathology-North, John Hunter Hospital, New Lambton Heights, NSW 2305, Australia; 10 Hunter Cancer Research Alliance (HCRA), Hunter Medical Research Institute (HMRI), New Lambton Heights, NSW 2305, Australia

**Keywords:** multiple sclerosis (MS), genetic wide association study (GWAS), single nucleotide polymorphisms (SNPs), natural killer cells, human leukocyte antigen (HLA) complex, natural killer gene complex (NKC), leukocyte receptor complex (LRC), multi-variate regression analysis, elastic net, gene–gene interaction

## Abstract

Conventional genome-wide association studies (GWASs) of complex traits, such as Multiple Sclerosis (MS), are reliant on per-SNP *p*-values and are therefore heavily burdened by multiple testing correction. Thus, in order to detect more subtle alterations, ever increasing sample sizes are required, while ignoring potentially valuable information that is readily available in existing datasets. To overcome this, we used penalised regression incorporating elastic net with a stability selection method by iterative subsampling to detect the potential interaction of loci with MS risk. Through re-analysis of the ANZgene dataset (1617 cases and 1988 controls) and an IMSGC dataset as a replication cohort (1313 cases and 1458 controls), we identified new association signals for MS predisposition, including SNPs above and below conventional significance thresholds while targeting two natural killer receptor loci and the well-established HLA loci. For example, rs2844482 (98.1% iterations), otherwise ignored by conventional statistics (*p* = 0.673) in the same dataset, was independently strongly associated with MS in another GWAS that required more than 40 times the number of cases (~45 K). Further comparison of our hits to those present in a large-scale meta-analysis, confirmed that the majority of SNPs identified by the elastic net model reached conventional statistical GWAS thresholds (*p* < 5 × 10^−8^) in this much larger dataset. Moreover, we found that gene variants involved in oxidative stress, in addition to innate immunity, were associated with MS. Overall, this study highlights the benefit of using more advanced statistical methods to (re-)analyse subtle genetic variation among loci that have a biological basis for their contribution to disease risk.

## 1. Introduction

Multiple sclerosis (MS) is an autoimmune disease driven by a combination of genetic predisposition and environmental factors [1] including reduced levels of vitamin D [2], smoking [3] and viral infections, such as Epstein–Barr virus (EBV) and cytomegalovirus (CMV) [4]. The most consistent and strongest genetic association with MS is conferred across the human leukocyte antigen (HLA) complex [5], specifically by the HLA-DRB1*1501 variant with an average odds ratio of 3.08 [6]. The HLA loci is involved in distinguishing ‘self’ from ’non-self’ through the expression of proteins involved in antigen processing and presentation, primarily interacting with CD4+ and CD8+ T cells [7]. The high degree of sequence variation across the HLA region makes it difficult to characterise the genetic architecture of this region in MS [5,8,9]. Furthermore, it is highly likely that interactions with loci outside of the HLA locus contribute conjoint effects to disease causation.

Beyond binding antigens and interacting directly with CD8+ T lymphocytes, HLA class I proteins also engage with natural killer (NK) cell receptors to either promote or inhibit their function [8]. Unlike CD8+ T cells, NK cells can lyse a target cell without priming depending on the balance of inhibitory and activating NK receptors. NK receptors are encoded within the Natural Killer gene complex (NKC) [10] and Leukocyte receptor complex (LRC) [11], and act in combination with their respective HLA ligands on the target cell [12]. Indeed, NK cells have been increasingly implicated in MS aetiology and attributed to some treatment success [13,14,15,16]. However, NK cells in autoimmune diseases are reported to have duplicitous roles [17,18]; activated NK cells have been shown to be able to kill autologous and heterologous oligodendrocytes in vitro [19], present in acute inflammatory lesions [20] and expansion and reduction of specific NK subsets (reviewed in Chanvillard et al. [14]). Therefore, determining if there is a genetic predisposition for altered NK cells involving certain receptor–ligand interactions may help guide future studies in the aetiology of MS.

The genome-wide analysis study (GWAS) design has enabled the discovery of more than 200 non-HLA loci associated with MS [21], explaining 20–30% of the total disease risk [22]. However, since GWASs can involve testing millions of single nucleotide polymorphisms (SNPs) in a one-test-per-SNP manner, they suffer from high type I error rates. For this reason, a high significance threshold of α = 5 × 10^−8^ has routinely been adopted to minimise type I errors as a result of multiple testing [23]. This analytical approach can inadvertently increase the type II error rate by discarding SNPs with true, albeit small, effect sizes. Methods have been developed to deal with SNPs that are suggestively significant (i.e., 5 × 10^−8^ < *p* ≤ 0.05). For example, Baranzini et al. [24] developed a pathway and network-based analysis to show that additional genes and biological pathways specific to MS could be identified (compared to GWAS data from other diseases) through the consideration of all disease ‘suggestive’ SNPs. However, even this method remains reliant on an initial one-SNP-at-a-time p-value-based assessment. Thus, more appropriate methods are required to mine GWAS datasets to uncover potentially important genetic and biological insights into MS.

Penalized (or shrinkage) regression methods can overcome the ‘curse of dimensionality’ problem of GWASs by including a constraint (or penalty) in the analysis to diminish the coefficients of less informative SNPs toward zero. With appropriate tuning parameters, this approach can be applied to GWAS datasets to detect a panel of SNPs predictive of disease outcome without generating individual P-values and the need to meet stringent significance thresholds. The GLMNet package [25] was developed to apply efficient penalized regression procedures that can fit the entire least absolute shrinkage and selection operator (lasso) or elastic-net regularization path for various regression models. While elastic net has been available since 2005 [26], and the R package GLMNet since 2009 [25], there are relatively few GWASs that have moved beyond evaluating their models in simulated and publicly available datasets. Studies that have utilised penalised regression for GWAS have tended to focus on feature selection, opting to use lasso regression to determine a subset of associated variables. For example, Mavaddat et al. [27] successfully applied lasso to improve the polygenic risk score and associated odds ratio (OR) in breast cancer, and Wu et al. [28] for coeliac disease, despite the overwhelming signal conferred by the HLA locus, identified several additional non-HLA loci that remained undetected by conventional GWAS analysis. 

This has led to development of more computationally efficient methods to fit lasso to high dimensionality datasets, including specifically for genotype data such as AUTOLASSO [29] and snpnet [30]. However, the lasso method is best suited to feature selection rather than discovering the biological underpinning of genetic associations and potential interactions due to the loss of correlated variables. Waldman et al. [31] evaluated the use of lasso and elastic net in simulated and real-world GWAS data and identified an alpha set at 0.1 (tuning parameter for elastic net determining ‘how close’ it penalises similar to lasso—see methods) as the best compromise between false positives and maximising the number of correct variables selected. This was the alpha setting applied in the current study, as we aimed to capture interacting SNPs by avoiding the loss of correlated variables. Benton et al. [32] successfully detected differentially methylated regions (DMRs), which relied on the identification of correlated CpGs, using an elastic net set toward ridge regression. Therefore, our study is the first to utilise an elastic net framework on real-world MS GWAS data with the aim of overcoming initial p-value thresholds to capture and evaluate the complex genetic architecture and associated biological basis of MS. These findings were achieved using a relatively small cohort of patients, by the incorporation of novel adaptations onto elastic net. This includes a similar approach to Meinshausen and Bühlmann [33], but on elastic net instead of lasso, whereby we perform ‘stability selection’ via iterative subsampling (with replacement).

In the current study, we have adapted elastic net to re-analyse previous MS-GWAS datasets to better understand the genetic signature in MS across the HLA and NK receptor loci. We include a bootstrap wrapper to assign individual SNP probabilities, as well as a weighting algorithm that tempers the strong signals contributed by HLA whilst reducing unnecessary loss of weaker signal at NK loci. This alternate approach to analysing GWAS data has allowed us to reveal new insights into the relationship of HLA and NK receptor loci in MS and confirm findings recently revealed in a much larger GWAS [34].

## 2. Materials and Methods

### 2.1. Overview of Analysis Pipeline

**Figure 1:**
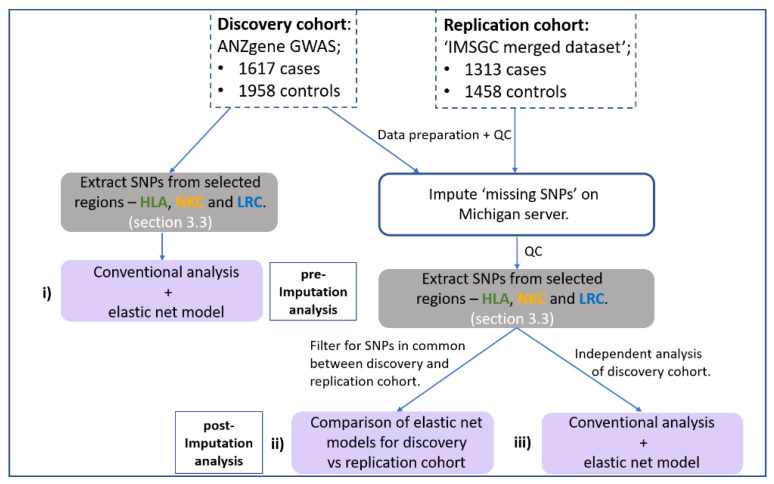
Flow chart outlining the major steps within the analysis pipeline. Two MS GWAS have been re-analysed. (**i**) First, the HLA and NK receptor loci were extracted from the pre-imputed discovery cohort and both a conventional GWAS and elastic net analysis performed and compared. Haploview [35] was used to determine if the SNPs identified by elastic net were in disequilibrium. Secondly, both the discovery and replication cohort were imputed using the Michigan server with resulting SNPs subject to a stringent imputation quality control (QC) threshold (R^2^ < 0.8) and extraction of SNPs from the same HLA and NK receptor genetic boundaries. (**ii**) Then, an overlapping elastic net analysis was performed on SNPs in common between the discovery and replication cohort that met imputation QC threshold. (**iii**) Thirdly, an in-depth independent analysis on the imputed discovery cohort was performed to maximise coverage of high-quality imputed SNPs. Grey boxes indicate the stage of SNP extraction across the three regions, and purple boxes indicate the three different combinations of datasets and SNPs being analysed.

This study implemented a gene-centric analysis of previously reported MS-GWAS data focusing on the HLA, NKC and LRC regions. Figure 1 illustrates the workflow for the major steps of the analysis for this study. The general aim was to identify whether SNPs at the NK receptor loci (NKC and LRC) act in combination with HLA SNPS to influence MS risk. Considering the limitations of standard GWAS analysis, which employs a ‘one-at-a-time’ approach to analyse each SNP, our approach used a generalised linear regression model with stability selection to consider all SNPs within a single model. This was achieved using an elastic net penalty set toward a ridge-regression with stability selection via iterative subsampling, henceforth referred to as the ‘elastic net model’. (See Section 2.6 below for more detail). The elastic net model was chosen because it can capture correlated and potentially interacting SNPs while removing those that do not contribute any predictive value to the outcome (disease status). The addition of iterative subsampling was performed to further reduce potential overfitting and assign a relative degree of importance (probability) to associated SNPs/genomic regions identified. Therefore, the elastic net model was implemented to achieve three specific aims:

(I) detect any additional signal across the three loci (HLA, NKC and LRC) that standard GWAS analysis missed (Figure 1i).

(II) identify SNPs with a similar signal strength across two imputed GWAS cohorts and determine which biological pathways these SNPs are associated with (Figure 1ii).

(III) determine the gene (or intergenic) region associated with disease risk when accounting for the combined signal strength of multiple SNPs representing the same region (Figure 1iii).

### 2.2. Datasets and QC

Table 1 briefly outlines the demographics and sample size for both datasets used. The ANZgene GWAS dataset was used as a discovery cohort [36] with one case removed due to an ambiguous phenotype leaving 1211 relapse onset (RO [relapse-remitting + secondary progressive MS]), 406 progressive onset (PO) MS and 1988 controls. 

Publicly available data from the IMSGC were obtained from the database Genotypes and Phenotypes (dbGaP) for phs000275 [37], phs000139 [38] and phs000171 [38] as a replication cohort containing an unknown mix of MS subtypes. The national (UK) blood service (NBS) control cohort, obtained from the Wellcome Trust Case Control Consortium, which matches ‘the distribution of the samples in the 1958 British Birth Cohort’ [39] (the control cohort used in the ANZgene dataset). Datasets were updated to Hg19 coordinate build [40] and merged on common SNPs among the three platforms using custom scripts in R [41], and the same quality control (QC) threshold used for the ANZgene dataset was applied to the merged IMSGC dataset (henceforth referred to as the ‘IMSGC dataset’) using plink v1.9 [42,43]. Both the discovery ANZgene dataset [36] and the IMSGC dataset [37,38], used in this study, targeted and QC’d their studies toward case-control samples of European descent.

Access to these datasets was obtained following and under approval by the institutional review board of Hunter New England Human Research Ethics Committee (2019/ETH12346).

### 2.3. Imputation

Imputation was performed on entire chromosomes (6, 12 and 19) using Minimac3 on the Michigan server [44] with the human reference consortium [45] as a reference panel, phasing with Eagle (v.2.3) [46] and the European population selected for QC. Initial data preparation was performed as directed by the Michigan server. Imputed SNPs for both cohorts were subject to a stringent high-quality imputation threshold using the minimac3 estimated value of the squared correlation between imputed genotypes and true, unobserved genotypes set at R^2^ ≥ 0.8 [47]. This filtering was performed in plink converted to binary plink files and SNPs extracted from the selected regions. Imputation of SNPs was performed to maximise the number of variables that could be analysed for both datasets, and because one caveat of elastic net is that it cannot ‘handle’ missing data.

### 2.4. SNP Boundary Selection and Extraction

The hg19 coordinate build was used for all datasets and the USCS genome browser [48] was utilised to ensure all genes targeted within the given regions were captured. The boundaries for the HLA, NKC and LRC were based on a combination of the Genome Reference Consortium (GRC) website [49] and published literature [10,11,12], which were extracted using plink and corresponded to the following regions (see supplementary files ‘additional file S2’ for USCS exports):

HLA—Chr6:28,467,000-33,458,000 (GPX6-to-SYNGAP1).

NKC—Chr12:9,737,870-10,762,434 (KLRB1-to-KLRAP1).

LRC—Chr19:54,534,080-55,559,632 (VSMT1-to-GP6).

Only SNPs with complete genotype information (for all individuals) were selected because analysis by elastic net cannot be performed using incomplete (i.e., missing) data.

### 2.5. Standard Association Testing

The extracted regions were subject to Fisher’s exact testing using plink. Multiple testing correction was applied using both the gold standard GWAS Bonferroni adjustment (*p*-value < 5 × 10^−8^) and at a relaxed threshold relative to the number of variants analysed. Visualisation of ‘SNP significance’ by Manhattan plots was created using −log10 transformation of p-values in the ‘qqman’ package [50] in R.

### 2.6. ‘Elastic Net Model’ Optimisation with Stability Selection via BootNet (Iterative Subsampling)

GLMNet [25] is an R package that fits a generalized linear model via penalized maximum likelihood. An alpha of 0.1 was applied to all tests to perform an elastic-net penalty that tended towards a ridge model. An alpha set towards a ridge model (α = 0) was favoured over a least absolute shrinkage and selection operator (lasso) penalty (α = 1) to capture and avoid losing SNPs that may have a correlated disease association and may also be in linkage disequilibrium (LD) with neighbouring or long-distant SNPs (inter and intra-chromosomal). Feature selection is possible using lasso modelling (L1 regularisation), which shrinks less associated variable’s coefficients to zero removing them from the model. However, for associated variables that are correlated, otherwise known as multicollinearity (such as SNPs in LD), lasso tends to randomly drop one and keep the other thereby reducing model complexity. Therefore, lasso is ideal for biomarker selection by retaining only the most strongly associated/predictive variables. In contrast, ridge regression (L2 regularisation) will shrink less correlated variables toward but never reaching zero, thus keeping all variables within the model. Meanwhile, elastic net was created to overcome the limitations in both these algorithms by incorporating both L1 and L2 penalties, but to an adjustable degree. Furthermore, an alpha of 0.1 has previously been shown in simulated and real-world GWAS data to be optimal in capturing maximal true positives while minimising type I error, compared to lasso and other alpha levels [31].

The second parameter that requires tuning is λ/lambda (the degree of penalty applied to the model) to minimise the predicted mean-square error (MSE) obtained from cross-validation (cv) by GLMNet. This was set by stabilising the lambda.min value returned from cv by GLMNet with the default 10-folds. Since each time cv by GLMNet is run the data is randomly split into 10 folds, a slightly different ‘optimal lambda’ can be observed. Therefore, the average lambda.min was obtained from repeating cv 20 times. Lambda.min was chosen over lambda.1se (largest lambda within 1 standard error of the MSE) as we aimed to maximise the number of variables identified, and the additional stabilisation wrapper (described below) was utilised instead to further reduce potential type I error and assign relative SNP importance.

A custom ‘stability selection wrapper’ (called BootNet) was then applied to GLMNet using the lambda.min obtained from the prior cv procedure (and α = 0.1). In this context, the ‘wrapper’ is a code built on top of GLMNet that splits the data prior to running the algorithm (subsampling with replacement), identifies all the variants with a non-zero coefficient (retained within the model) and iterates (repeats) the procedure a select number of times. Therefore, this could be considered as ‘elastic net stability selection by iterative subsampling’ (with replacement). We performed 3000 iterations with 66% subsampling with replacement (of sample/case-control groups) to produce a table of SNPs that were retained/ ‘selected’ by elastic net with the number of times each SNP was identified and converted to percentage i.e., if SNP1 was identified 2400 times, this corresponds to 80% iterations. Thus, the term ‘elastic net model’ will be used to refer to the optimised algorithm with stability selection. A link to the code for this GLMNet stability selection procedure is made available below.

Results were visualised in R, with ‘Manhattan-inspired plots’ showing the percentage of times (iterations) each SNP was identified by the elastic net model against their genomic base position (bp).

### 2.7. Replication Analysis Using SNPs in Common

To further reduce any interpretation error from potential over-fitting, elastic net analysis was performed on both the ANZgene and IMSGC datasets independently but restricted to SNPs common to both datasets and met the imputed QC threshold (R^2^ ≥ 0.8). A combined false discovery rate (FDR) for each SNP was created by multiplying (1 − (number of iterations the SNP was selected/total iterations [3000])) from both cohorts. An absolute cut-off was set, removing any SNP below 30% iterations in either cohort. This was to prevent any SNP with a high signal (above 90% iterations) in only one cohort automatically being identified. For any SNP reaching 100% iterations, a nominal value of 1 less than the total number of iterations was used (i.e., 2999/3000) to enable ranking in relation to the number of iterations in the reciprocal cohort and were denoted with an Asterix. SNPs were considered highly robust and replicated (in common to both GWASs), with a combined FDR ≤ 0.1. This more stringent FDR value (in comparison to the initial 70% iteration threshold utilised for the preliminary analysis) was selected to provide a robust set of SNPs in common to both cohorts, while still providing flexibility, as the strength of signal from both cohorts was considered, i.e., so that SNPs with both a strong and moderate-strong signal in common to both cohorts can be identified, while ignoring SNPs with a strong signal in only one cohort.

Mapping of identified SNPs was performed using Kaviar [51] to obtain rsIDs as an input for dbSNP [52] and variant effect predictor (VEP) [40] to assign associated genes and their functions. Gene enrichment analysis [53,54,55] within ToppFun was performed on the resulting gene list with an adjusted FDR (Benjamini–Hochberg [B & H] ≤ 0.05).

### 2.8. Independent Analysis of the Discovery Cohort

All SNPs that met the stringent imputation QC threshold (R^2^ ≥ 0.8) for the ANZgene dataset were analysed by elastic net model (methods 2.6), independently. In addition to determining the percentage iterations for each SNP, the contribution of signal strength (iterations) for all SNPs within genomic boundaries (gene and intergenic regions) was evaluated to better understand the contribution of subtle genetic variations amongst larger signals. This was achieved by setting boundaries for each gene obtained from the USCS table browser—hg19 format [56]. Each intergenic stretch of interest was labelled according to the gene directly up or downstream, i.e., ‘INT (gene1_gene2)’. All SNPs identified by elastic net were then assigned to their specific gene or intergenic region according to their bp coordinates and the sum of iterations for each region calculated. Each genetic region identified was plotted showing the percentage of iterations for the given gene relative to the total number of iterations for all SNPs.

Static and interactive circos plots were created to compare the one-at-a-time P-value based Manhattan plot in a circularised format against the elastic net model for (1) percentage iterations for each SNP and (2) the contribution of iterations for all SNPs within the designated genetic boundaries. This was created with custom scripts using the R package ‘BioCircos’ [57,58]. Interactive plots are accessible as additional files and coded in html. We recommend viewing these in the web browser Chrome. These are intended to allow personal interrogation of the results and regions studied. Access to these plots is made available through the GitHub page: https://sburnard.github.io/Elastic_Net_MS_GWAS_paper_data/.

### 2.9. Haploview Analysis

Plink 1.9 was used to convert binary HLA genotype data for haplotype analysis by Haploview v4.2 [35] to reveal SNPs in linkage-disequilibrium (LD). When SNPs (at two or more loci) are in LD, they are deemed not to occur at random (not at equilibrium) in the studied population and may be ‘linked’. Both LD measurements D’ and R^2^ were considered. D’ is the difference between the observed and expected haplotype frequency (D’ = 1.0 is ‘complete disequilibrium’). R^2^, is the correlation between the pair of SNPs and is susceptible to alteration (reduced) when SNPs have different minor allele frequencies (MAFs). Therefore, SNPs can be found to be in LD (high D’) but still have a low R^2^ value. SNPs were deemed to be in LD and co-inherited (also referred to as ‘proxy SNPs’) if D’ ≥ 0.9 and R^2^ ≥ 0.8. 

The purpose being to identify potential independent SNP associations with MS and to add confidence in SNPs identified reaching the lower end of our bootstrapping threshold (70–80%). 

### 2.10. Epistasis Analysis

An exploratory epistasis analysis was performed on two different sets of SNPs and two different datasets, considering only inter-chromosomal SNPs; (1) the set of SNPs identified by elastic net analysis found to be in common to both the ANZgene and IMSGC dataset using a single combined ANZgene + IMSGC dataset, and (2) SNPs ≥70% iterations from the independent elastic net analysis of the discovery cohort using the same dataset. Plink 1.9 was used to both remove/ ‘prune’ SNPs in perfect LD (r^2^ >0.9999) *(--indep-pairwise 300 1 0.999*) and to perform epistasis analysis (*--epi1 0.05 --epistasis*). 

### 2.11. Code Availability

The code used for elastic net stabilisation (BootNet) can be found on GitHub (https://github.com/sirselim/bootNet) and archived in zenodo [59]. Access to all the associated figures and tables for this paper will also be made available on GitHub (including the interactive plots): https://github.com/SBurnard/Elastic_Net_MS_GWAS_paper_data.

## 3. Results

### 3.1. Re-analysis of the ANZgene GWAS by Elastic Net Identifies SNPs above and below Conventional p-Value Thresholds

A preliminary comparison of analytical approaches was performed using the ANZgene GWAS dataset as a discovery cohort with SNPs extracted from the NK receptor loci (NKC and LRC) and HLA region that were directly genotyped (see methods for analysis pipeline). Specifically, 1617 cases and 1958 controls yielded complete genotype data for 1047, 137 and 122 SNPs from the HLA, NKC and LRC regions, respectively (see Table 1 for a summary of the SNPs available for both cohorts). 

**Figure 2:**
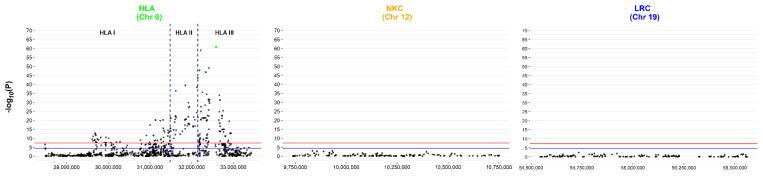
Conventional GWAS case vs. control analysis of the discovery dataset reveals no hits outside of the HLA loci. Manhattan plot for the HLA, NKC and LRC regions, using the log10 transformed *P* value from Fisher’s Exact testing against base position for the pre-imputed ANZgene dataset. The HLA loci contains SNPs reaching the gold standard GWAS Bonferroni correction threshold of 5 × 10^−8^ (red line), with two distinct peaks across the region. In contrast, neither the NKC nor the LRC loci contained SNPs that met the relaxed Bonferroni correction of 4 × 10^−5^ (blue line) accounting for the number of SNPs analysed. The SNP highlighted in green is the tag SNP for the drb15 haplotype.

Figure 2 shows the Manhattan plot created from the extracted SNPs. Only the HLA region contained SNPs reaching significance when using either the ‘gold standard’ GWAS Bonferroni correction threshold of 5 × 10^−8^ (red line) or with a relaxed threshold of 4 × 10^−5^ (blue line), i.e., accounting for the number of SNPs analysed within this targeted analysis. As previously reported by ANZgene [36], the tag-SNP for the HLA-drb15 haplotype, rs9271366, was the most significant (highlighted in green). There are two distinct peaks in the HLA region (Figure 2), the largest encompassing genes across HLA-class I, II and III with the apex in class II (rs9271366). The second region of interest, albeit with lower *p* values, is still prominent and located in class I, with the apex of the signal centred upstream of *MOG* and downstream of *HLA-F*. Table 2 highlights the 5 SNPs with the lowest *p*-values for each region. In terms of the NKC and LRC loci, even if the *p*-value thresholds are adjusted independently (accounting for the number of variables at a loci level), no SNP reached significance when corrected for multiple testing.

However, both the NKC and LRC loci contain SNPs that would be considered ‘statistically significant’ if each SNP were independently tested (i.e., *p*-value < 0.05) and thus may represent more subtle, yet biologically important, MS loci. For this reason, penalised regression using elastic net was employed, which takes into account the effect of all variables (SNPs in this case) within a single association model. Furthermore, using an elastic net with an alpha level set towards ridge regression (α = 0.1) served to increase the likelihood of capturing correlated SNPs associated with disease outcome, such as SNPs in LD or the interaction of SNPs within and between regions (e.g., between NK receptor loci and HLA loci). After applying elastic net with stability selection by iterative subsampling and setting an initial cut-off at 70% iterations, a panel of SNPs localising to all three regions were identified (Table 3), implicating a more comprehensive set of genomic differences that contribute to MS risk.

Not surprisingly, the majority of strongly associated SNPs identified by the elastic net model were from the HLA loci (24 SNPs ≥ 90% iterations). However, at least one SNP in both the NKC and LRC loci also reached over 90% iterations. For all three loci, only four of the five SNPs with the lowest p-value (Table 2) reached above the 70% threshold (Table 3, bold*), indicating that the elastic net model did not simply select SNPs with the lowest possible *p*-values and may also provide some filtering for those most correlated with disease. Furthermore, the SNP that reached the highest percentage of iterations in the NKC loci (rs10845080, 93.3%, *p* = 3.57 × 10^−3^) was not the SNP with the lowest p-value from that region (rs11053043, 82.7%, *p* = 9.91 × 10^−4^). The elastic net model also identified SNPs that would not meet any loose conventional threshold (*p* ≤ 0.05) in not only the LRC and NKC loci, but also the HLA region such as rs2844482 in *lymphotoxin alpha (LTA)* (98.1%, *p* = 0.673). This was unexpected, since the HLA loci contains an abundance of SNPs that already meet conventional and GWAS statistical significance (Figure 2). As expected, the HLA-DR15 tag-SNP rs9271366 was unequivocally associated with MS risk (Table 3). Elastic net identified a further 5 SNPs within the HLA loci that were also called in 100% of iterations. These SNPs are located within or immediately downstream of *HLA-DRA*, *HLA-DRB9*, *HCG23* and *c6orf10*.

**Figure 3:**
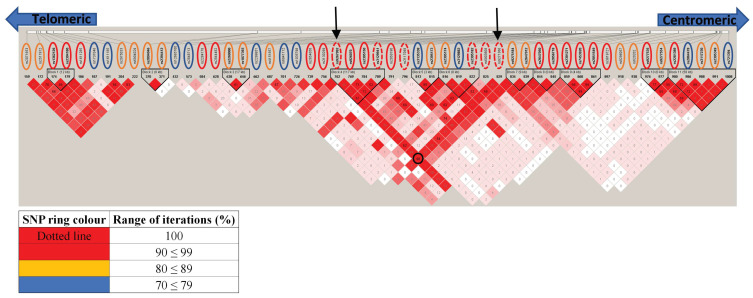
Haplotype structure of the HLA SNPs identified by the elastic net model (Table 2) using haploview. The rsIDs are given at the top of the display, with the coloured rings representing the following range of iterations they were selected by the elastic net model: blue (70 ≤ 79%), orange (80 ≤ 89%), red (90 ≤ 99%) and red dotted (100%). For each SNP, their corresponding r^2^ value are given within each diamond (shown as 0–100, which is equivalent to 0.00–1.0) and red shading indicating strength of D’ between SNPs intersecting diagonally (see Figure S1 for the individual r^2^ and D’ plots). As expected, there is a complex underlying LD structure across the HLA region with 11 blocks of inherited SNPs predicted by haploview, consisting of 2–5 SNPs each. Of the 12 SNPs that were identified above 98% iterations from elastic net analysis, only rs9267992 and rs9271366 (black arrows meeting at the black circle) were determined to be in strong LD and co-inherited (r^2^ ≥ 0.7 and D’ ≥ 0.8). These two SNPs also flank a central set of SNPs (encompassing blocks 4, 5 and 6), both with a relatively high level of LD for all SNPs located between them (see supplemental notes for additional comments).

Given the known and extensive LD across the HLA locus and because the elastic net model applied was designed to capture correlated variables (which could include both interacting SNPs and those in LD), haplotype block analysis was used to distinguish SNPs in LD from potential independent associations and asses the credence for the threshold set (≥ 70% iterations). Using the SNPs that met our elastic net model threshold (Table 3), Figure 3 illustrates the complex haplotypic structure across HLA in the ANZGene cohort with 11 haplotype blocks detected, containing 2–5 SNPs within each block. Most haplotype blocks identified (7 out of 11) included at least one SNP that reached above 90% iterations (Figure 3) with each haplotype block most likely representative of an independent genetic association. Haplotype block 4, consisted of four SNPs above 90% iterations, two of which reached 100% iterations, suggesting a strong and common association with MS. Haplotype block 6 included three SNPs that covered a range of iterations (rs2395182; 100%, rs2395174; 87.0%, and rs3129882; 78%). This block maps onto *HLA-DRA* with rs2395182 which is already associated with *HLA-DR15* status and an established risk factor for MS. 

Haplotype block 11 also encompassed SNPs covering a range of iterations, including rs721394, identified by elastic net that would not have met any conventional threshold (71% iterations, *p* = 0.417). Therefore, the identification of some of these SNPs above conventional thresholds could be explained by LD, such as rs9275184 (88.1% iterations, *p* = 0.529) found to be in LD with rs2647012 (88.2% iterations, *p* =1.1 × 10^34^) and forming haplotype block 7. As SNPs can be in LD and ‘inherited together’ but not necessarily at the same frequency, both LD scores D’ and r^2^ were considered (Figure 3 and Figure S1). Meanwhile, a ‘proxy SNP’ more meaningfully refers to SNPs that are co-inherited (Table S1). For rs9275184 and rs2647012 their LD score (D’ = 1 and r^2^ = 0.08) reflects their minor allele frequencies (MAFs) of <0.09 and 0.4, respectively (see additional data). Therefore, since these SNPs were found to be in LD, it is very likely that with an increased sample size that rs9275184 would become ‘significant’ by conventional comparison, but our elastic net model has circumvented this need for an increased sample size.

There were a number of SNPs not in LD with any other HLA associated SNP that may indicate unique independently associated loci that were not previously revealed. For example, rs12665700 (72% iterations, *p* = 0.662), rs2844482, (98.1% iterations, *p* = 0.673), rs2299851 (74.8%, *p* = 2.32 × 10^−3^) and rs3819721 (92.7%, *p* = 7.7 × 10^−12^) mapped to *MUC22, LTA, MSH5* and *TAP2*, respectively, and were not in LD with any other HLA SNPs identified by elastic net (Figure 3). Overall, these results corroborate the inclusion of SNPs that pass the 70% iteration threshold in revealing key signals and with the percentage of iterations possibly indicative of their relative levels of importance. (see the Supplemental note in ‘Supplemental Data’ for a detailed interrogation of haplotype analysis.)

### 3.2. Replication Analysis Using SNPs Common to Both the Discovery and Replication Cohort

To further reduce the risk of potential overfitting that can cause false positive associations, the use of, and comparison to, a replication cohort was undertaken. After filtering both cohorts for genotyped and imputed SNPs with high accuracy (R^2^ ≥ 0.8) within the selected boundaries (see methods); 2359, 2872 and 520 SNPs were common between the two cohorts for the HLA, NKC and LRC loci, respectively (Table 1).

Only the NKC loci had a good distribution of SNPs across the whole region. This was primarily due to the loss of SNPs when merging the replication dataset from four different platforms (see methods), resulting in a lower yield of high-quality imputed SNPs. Following elastic net analysis for both cohorts independently, multiple SNPs for all three loci in either cohort exceeded 70% iterations (Figure S2). Focused signal peaks (reaching toward 100% iterations) were observed in the HLA loci for both cohorts, with commonality of peaks between cohorts located towards the centromere, in HLA class II. There were also distinct peaks observed in the LRC loci for both cohorts, but with a greater overall signal strength seen in the replication cohort and less clear commonality between cohorts. The regions between the peaks mostly represented missing data, rather than unselected SNPs by elastic net. The NKC loci produced a strong signal across the entire region for both cohorts. To discern SNPs that were of interest and of similar importance across the two cohorts, a combined FDR value (≤0.1) was evaluated. This revealed 69 SNPs common (Table S2) to both cohorts. For those SNPs that aligned with a corresponding gene in dbSNP (Table 4), the resulting gene list was then used for functional enrichment analysis by ToppFUN (Table 5). 

In total, 52 HLA, 15 NKC and 2 LRC SNPs were identified to be the most robust of the studied loci when considering both the discovery and replication cohorts (Table S2). One third of the SNPs (23 out of 69) were in intergenic regions, while the remainder corresponded to 16 HLA and 12 NKC genes (Table 4). Several HLA genes had three or more associated SNPs, including *GPX6*, *HLA-DOB*, *TAP2*, *C2* and *TNXB*. Additionally, non-coding transcript variants for *GPX5* and *LINC02390*, as well as missense SNPs for *GPX6*, *LTA* and *TNXB*, were identified. In total, 10 SNPs mapped to the gene *TNXB,* which is known to have multiple transcript variants and overlaps at its 5’ and 3’ ends with *CREBL1* and *CYP12A2*, respectively.

Consistent with the autoimmune and antigen driven association with MS, molecular functions and pathways involved in antigen/ protein binding, processing and presentation were identified (Table 5). MHC class I binding was overrepresented with multiple SNPs having identified *TAP2* and *TAP1* (Table 3). While not all the receptors within the NKC loci are exclusive to NK cells, gene ontology (Biological Process) ‘top hits’ indicated the involvement of natural killer cell activity rather than any other leukocyte subset (Table 5).

### 3.3. Independent Analysis of Discovery Cohort Provides Further Insight Due to Imputation

**Figure 4:**
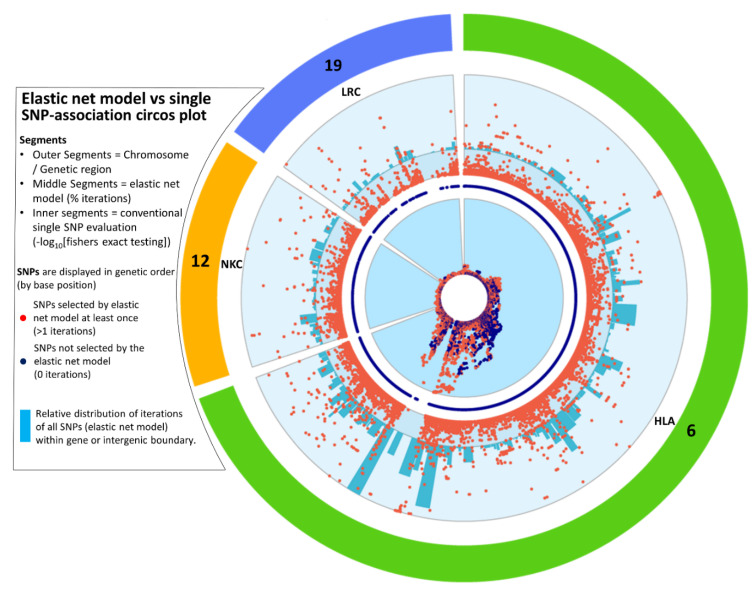
Circos plot for the imputed SNPs from the ANZgene dataset, extracted from the HLA (green), NKC (orange) and LRC (blue) loci on chromosomes 6, 12 and 19, respectively. Each dot represents a single SNP plotted clockwise in bp order (see Methods for region boundaries). This plot compares one-at-a-time SNP evaluation using *P* values against our elastic net model for the exact same set of SNPs. The innermost ring is the -log10 *P* values from Fisher’s Exact testing (a circularised Manhattan plot). The values range from 0 to the outer edge representing the lowest *P* value (65.3). SNPs in blue represent those that were never selected by elastic net with bootstrapping (0 out of 3000 iterations), while orange signifies SNPs that were selected by elastic net with stabilisation by iterative subsampling (≥1 out of 3000 iterations). The outer ring with orange dots, represents the percentage of iterations that each SNP was selected by elastic net with 3000 iterations, ranging from 0 to 100 at the outermost edge. The middle ring of blue SNPs indicates the position of SNPs that were never selected by elastic net. Each dot representing the elastic net result is aligned at the same degree as the dots representing the -log10 *P* value in the innermost circle. The outermost ring with blue bars represents the combined contribution of all the iterations of each SNP (from elastic net with bootstrapping) within each genomic boundary relative to the total number of iterations, given as a percentage. The scale ranges from zero to 4.17%, which is the largest value representing the intergenic region between HLA-DQB1 and HLA-DQA2.

An independent analysis of the imputed ANZgene data was performed to maximise the coverage of SNPs analysed across the three loci, while maintaining a high imputation accuracy threshold (R^2^ ≥ 0.8). This enabled the interrogation of 54541 HLA, 3790 NKC and 1576 LRC SNPs (Table S1). Analysis by elastic net of these more densely covered regions highlights the complex genetic architecture contributing to MS, particularly across the HLA region (Figure 4). Distinct peaks were observed across all three loci with both the NKC and LRC containing peaks approaching 100% iterations (see Supplemental for individual interactive plots of the HLA, NKC and LRC loci). When directly comparing the p-value based single SNP association testing (Figure 4, inner ring) to the elastic net model output (Figure 4, outer ring), we were able to identify additional SNPs that are biologically relevant and associated with disease (Figure 4, orange dots). Furthermore, elastic net analysis also discriminated between those SNPs whose *p*-values were suggestive of association-based significance thresholds (by GWAS analysis) that could be ignored (Figure 4, blue dots).

To take into consideration the contribution from multiple SNPs and ascribe signal strength to a gene or intergenic region, all SNPs identified by the elastic net model for the imputed ANZgene dataset were assigned by their bp to the genetic boundaries obtained from the USCS genome browser. For an unbiased interpretation of disease association, the percentage contribution of each genetic boundary was made relative to the total number of iterations across all SNPs (Supplemental file ‘additional file S2’). The blue bars in Figure 4 map this combined ‘signal’ from all identified SNPs to their respective gene or intergenic region across the HLA, NKC and LRC loci. 

The strongest genetic boundary signal for both NKC and LRC was a quarter of the greatest signal in HLA (Figure 4). However, it is important to note that the boundaries in the HLA are on average much larger than that of the NKC and LRC, as well the HLA containing a denser coverage of SNPs in this dataset. The strongest boundary signal for NKC was located in the intergenic region between *CD69* and *KLRF1* and the most prominent gene *CLEC2D* (Figure S3a). For the LRC, the strongest boundary signal was the intergenic region between the *LILRA1* and *LILRB1*, which was the region also identified in the replication analysis (Table 4), and the gene with the strongest overall signal was *LILRA1*, which was also the second most prominent signal within the LRC loci. The genetic boundary and individual SNP signals peaks within this ILT/LIR family, encompassing *LILRA1* to *LILRB4* (Figure S3b), encode receptors located predominantly on myeloid lineage cells and some NK and T cells. The most associated individual single SNP for the LRC was located in *VSTM1*.

As expected, the HLA region accounted for the highest proportion of total iterations (82.8%) covering ~5Mb and included 10 times as many SNPs compared to the NKC and LRC loci, which are both ~1Mb in size and account for 9.8% and 7.4% of the total number of iterations, respectively. The intergenic region *HLA-DQB1* to *HLA-DQA2* had the strongest signal of any region (4.17%), even though none of the SNPs within this boundary reached 100% iterations, while the intergenic region downstream of *HLA-DRA* contained all six individual SNPs reaching 100% iterations and was the fourth highest boundary (2.14%). This highlights the difference between only ranking regions by the iterations at an individual SNP level, compared to considering the number of iterations of all SNPs within a given region. A further seven boundaries contained at least one SNP above 99% iterations, with two located in HLA-class I and five located in HLA Class-II, mostly clustered around *HLA*-*DRA* and the boundary with HLA Class-III. This prominent cluster of signals in HLA Class II consisted of both individual SNP scores (Figure S4, black bars) and gene boundary scores (Figure S4, green bars). The six genes with the highest boundary scores were *c6orf10*, *HLA*-*F*-*AS1*, *HLA*-*DPB1*, *TRIM26*, *MUC22* and *NOTCH4*, while the highest representative SNP for each of the boundaries ranged from 54.3% for *TRIM26* to 99.3% for *HLA*-*F*-*AS1* (see supplementary files). For HLA class III, the regions bounded by *BAG6* and *GPANK1* both contain one SNP above 90% iterations. HLA class I is also a region of interest as there are distinct signals represented by both genetic boundary and individual SNP scores including a signal around the classical HLA Class-I receptor, *HLA-B* and another at *HLA-F-AS1* which overlaps with the non-classical HLA Class-I receptor, *HLA*-*F*. 

Overall, this elastic net boundary analysis highlights the need to consider signals from potentially subtle genetic regional variation (multiple SNPs) in concert with individual strongly associated SNPs that potentially may contribute to disease aetiology. However, the biological consequence of these signals still needs to be elucidated.

### 3.4. Epistasis Analysis

An exploratory epistasis analysis was performed on inter-chromosomal SNPs identified by the elastic net models to identify if any potentially interacting SNPs were identified. For the set of 69 SNPs identified from the replication analysis (Table 4 and Table S2), the discovery and replication cohort were merged and assessed. After pruning (removal of SNPs in near-perfect LD, 49 SNPs remained for the analysis. This identified several potential epistatic interactions between the HLA and NKC and LRC, but not between the NKC and LRC, for SNPs located in genes and intergenic regions (Table 6). Several potential epistatic interactions were detected for a SNP in the transcribed pseudogene *KLRA1P* and SNPs in the HLA loci, which could suggest the ubiquitously expressed but non-translated *KLRA1P* may have multiple genetic interactions that could influence MS. Similarly, an intergenic SNP between *LILRA1* and *LILRB1* also showed potential interaction with multiple SNPs in different genes from the HLA loci. 

Epistasis analysis was then performed using elastic net results from the independent discovery cohort analysis (Figure 4) due to the benefit of the improved coverage of SNPs across the three loci (Table 1). After pruning the 108 SNPs that reached above 70% iterations, 72 SNPs were assessed, which identified additional epistatic interactions (Table S3). Epistatic interactions between all three loci were identified with SNPs mapping to intergenic regions, intron variants, non-coding variants and synonymous variants. In the LRC, the same intergenic SNP between *LILRA1* and *LILRB1* identified in the replication epistasis analysis (Table 6) and an additional SNP in *LILRA1* showed an interaction with several HLA SNPs (Table S3) including the *HLA-DRA* synonymous variant rs3135391 (chr6:32410987), which previous studies have correlated with HLA-DRB1*1501 allele. 

### 3.5. Confirmation of Elastic Net Model Hits to Largest MS Meta-Analysis

**Figure 5:**
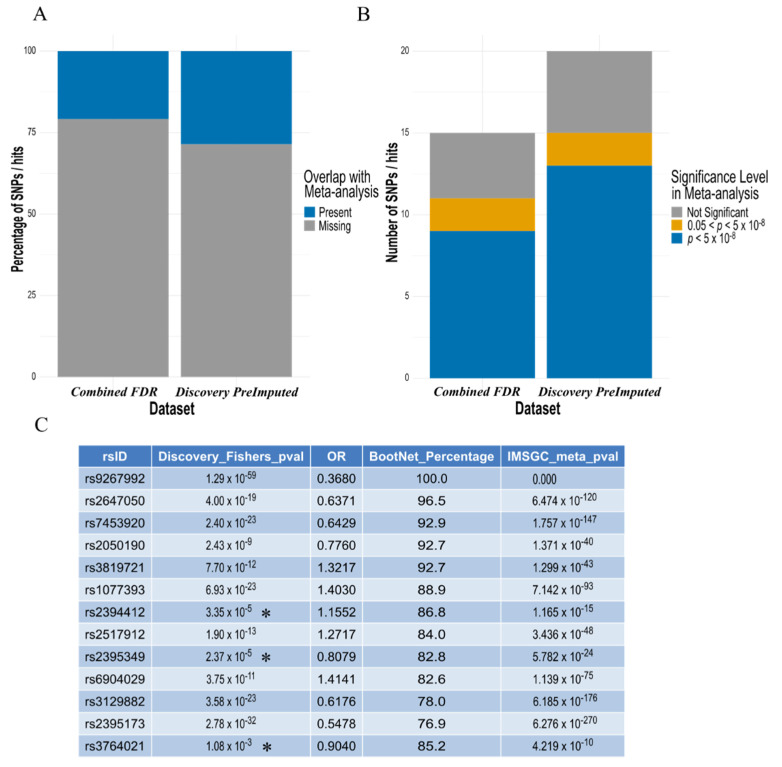
The majority of SNP ‘hits’ identified by the elastic net model, also present in the IMSGC meta-analysis, are found to be increasingly ‘significant’ by conventional methods in the largest MS dataset. (**A**) Around 25% of the hits identified by both the ‘combined FDR analysis’ (Table 4 and Table S2) and the pre-imputed discovery dataset were present in the IMSGC meta-analysis. (**B**) For the SNPs that were present in the meta-analysis, the majority were found to have *P* value below the conventionally adopted GWAS threshold (*p* < 5 × 10^−8^) in both analyses. (**C**) Comparison of the 13 SNPs found to reach GWAS level significance in the IMSGC meta-analysis with those identified by the elastic net model in the discovery dataset, confirmed all hits with increasing significance in the meta-analysis compared to the discovery *p* value. This also included at least three SNPs identified by the elastic net model with *p* values not reaching GWAS significance threshold in the discovery dataset (denoted with an Asterisk *).

Comparison of our hits from the pre-imputed discovery analysis (Table 2) and combined FDR ‘replication analysis’ (Table 4 and Table S2), to those present in the meta-analysis summary statistics from the most recent and largest IMSGC MS study (Figure 5a), confirmed the majority identified by the elastic net model reached conventional statistical GWAS thresholds (*p* < 5 × 10^−8^) in this much larger dataset (Figure 5b). Furthermore, at least three of these SNPs were not below this threshold in the discovery dataset, when identified by the elastic net model (Figure 5c). The full table comparing the original *p* values and our elastic net model results to the IMSGC meta-analysis can be found in Table S4.

## 4. Discussion

Overall, we present findings that support a genetic predisposition to MS across the HLA, NKC and LRC loci for MS while highlighting the need to re-evaluate GWAS data using statistical tools such as penalised regression that can handle thousands to millions of variables at once to provide biological insight into disease aetiology. We have shown there is a significant amount of information that has been untapped within existing GWAS data. While this approach has better implicated specific genetic signals contributing to MS susceptibility, the biological consequences remain to be confirmed. 

Arguably, the greatest issue with multiple testing correction, when relying on *p*-value-based statistical methodologies for large studies such as GWAS, is the extraordinary samples sizes required to overcome type II errors when trying to control for type I errors. The results presented herein argue that with a relatively modest GWAS sample size, the elastic net model was able to discriminate which SNPs were most likely to be associated with MS; for SNPs including those (i) above the conventional GWAS threshold (*p* < 5 × 10^−8^), (ii) of nominal or suggestive significance (5 × 10^−8^ < *p* < 0.05), and (iii) below conventional statistical threshold (*p* > 0.05), for all three loci studied. For example, our elastic net model for the pre-imputed discovery cohort identified SNPs in the NKC and LRC of nominal and borderline significance, while, for the HLA, at least four SNPs that reached above 70% iterations had an associated p-value above 0.4. This is particularly surprising, considering the amount of ‘highly significant’ p-value-based SNPs within the HLA loci (Figure 2). In particular, rs2844482 reached 98.1% iterations (Table 3) and maps to *LTA*, a gene previously associated with MS disease aetiology [60], including in terms of SNPs [61], methylation [62] and expression differences [63], and would be completely ignored by conventional statistical methods (*p* = 0.673). We confirmed this not to be in LD with any other SNPs (Figure 3). Thus, combined with the elastic net model result, this indicated a strong independent association with MS. The latest International Multiple Sclerosis Genetics Consortium (IMSGC) study, which is the largest MS study to date (>47K cases, and >68K controls), also identified rs2844482 as the fourth largest independent HLA risk factor MS (*p* = 7.13 × 10^−124^) [34]. This report by the IMSGC (2019) successfully confirmed and expanded, at a genome-wide level, the number of loci associated with MS and went further to assign likely biological association. This enabled the authors to implicate genetic alterations and pathways associated with both adaptive and innate immune cells, including NK cells. However, their methodology was still reliant on conventional p-values both as end discriminator, and as an initial screening tool; with any SNP not reaching ‘suggestive significance’ (*p* < 0.05) removed from any further analysis. Therefore, if we had adopted a prior *p*-value-based screening approach, this study would not have successfully detected many of the SNPs discussed.

A rationale for our elastic net model identifying SNPs when they are of borderline or below conventional significance, such as rs2844482, is the combination of elastic net being able to consider all variables at once with the addition of the stability selection wrapper which utilises subsampling. More specifically, these SNPs may be marginally overrepresented in combination with other disease relevant SNPs in our studied cohorts, and the ‘random’ subsampling of cases and controls for each iteration of elastic net further highlighted this disparity in lieu of a much larger sample size. Therefore, novel methods to study diseases are warranted, particularly for those that are rarer and unable to gather the sample sizes required by conventional GWAS. Nevertheless, new approaches would need to be devised to validate and provide confidence in the findings of the elastic net model when detecting SNPs below conventional statistical thresholds, especially when larger GWAS are unavailable for comparison. Fortunately, we were able to compare our ‘hits’ identified by the elastic net model to that of the summary stats from the latest, and largest IMSGC meta-analysis, which included up to 15 different GWASs (Figure 5). The ‘hits’ that were compared were from (1) the preliminary and pre-imputed discovery analysis (Table 3), which represent directly genotyped SNPs to ensure utmost confidence in the accuracy of SNPs included in the analysis, and (2) the combined FDR ‘replication analysis’ of the two datasets (Table 4 and Table S2), which represents a set of robustly MS associated SNPs, common across both independent cohorts (post-imputation). The majority of SNP hits that were also present in the IMSGC meta-analysis (Figure 5a), were found below conventional statistical GWAS thresholds (Figure 5b) with at least 3 SNPs in our discovery dataset originally below this threshold while identified by elastic net (Figure 5c). This provides additional confidence in the SNPs identified by the elastic net model in our modest sized GWAS datasets. Ideally, future studies would test and compare this analysis directly using individual genotype data from these larger scale studies, rather than using only summary statistics.

The use of penalised regression models for GWAS is not new, but it is under-appreciated/ utilised. There is also an apparent trade-off with the use of machine learning approaches prioritising feature selection for either prediction (of an outcome) or gaining biological insight. For example, Wei et al. 2009 used support vector machine (SVM) to improve the accuracy of type 1 diabetes disease risk prediction, developed and tested on three large scale GWAS datasets [64]. Furthermore, the authors also compared SVM to logistic regression with an L2 penalty (ridge regression) that was modified and developed by Park and Hastie [65] to aid gene–gene interaction, and showed the former was more accurate in this context. Interestingly, Wei et al. also utilised and assessed several *p* value-based pre-screening levels (*p* < 1 × 10^−8^, 1 × 10^−7^, 1 × 10^−6^, 1 × 10^−5^, 1 × 10^−4^ and 1× 10^−3^) prior to input into the model development and found that in some instance that the area under the ROC curve (AUC) improved for the L2 logistic regression models with increasing looser thresholds compared to the inverse effect for SVM [64]. More recently, Ghafouri-Fard et al. [66] published a preliminary report testing an artificial neural network (deep learning model) to predict MS disease risk, developed using a subset of SNPs (23 genotyped SNPs, across 11 genes) and a relatively small cohort (401 MS patients, and 390 controls), and reported a modest ROC AUC (69.67%). The inclusion of all SNPs across the genome would be the next logical step but may be computationally demanding with such an approach. The authors also utilised L2 regularisation (ridge regression) of hidden layers to ‘reduce over-fitting and enhance model generalisation’. Arloth et al. [67] in a modest sized cohort of MS and control data, applied a combination of deep learning (DeepSea) to identify ‘functional units’, which encapsulates SNPs with known regulatory effects of chromatin features (within 1000 bp from the SNPs present in the studied dataset) and treatments, leveraged from publicly available cell line experiment data. Similarly, they then utilise stability selection, but with L1 regularisation (lasso) to perform feature selection, and identify which of the SNPs from these ‘functional units’ were then associated with MS. They go further, to test the SNPs identified with publicly available cis-eQTL ci-meQTL, and cis-eQTM data, aiming to assign additional functional and biological importance. This latter approach is one that could be more readily and standardly adopted for all GWAS. 

Similar to our approach, the L2 penalised logistic regression model developed by Park and Hastie for gene–gene interaction with SNPs also validated their findings using a bootstrapping approach with a set lambda (identified by prior cross-validation) to assess the frequency each variable was selected and compared those with the highest frequencies to other studies [65]. In the meantime, there have been further developments of more computationally efficient implementation of these various algorithms, but the underlying statistical principles typically remain the same (each with their own strengths and weakness). Therefore, the most important principle remains to use the most appropriate tool for the aims and dataset being investigated, while still appreciating the potential weaknesses (and attempt to mitigate these where possible). Thus, since our aim was to aid biological insight by the identification of potentially interacting SNPs, we opted to use an elastic net model set towards ridge regression which has been previously shown in GWAS data to maximise variable selection and minimise false positives [31]; and has also been shown to capture correlated variables in methylation data [32].

In this study, we adopted two additional approaches to validate the findings by our elastic net model (which included stabilisation). Firstly, for our pre-imputed discovery cohort analysis, haplotype structure was interrogated with the use of LD plots providing some credence for the use and benefit of a 70% iteration threshold as an initial cut-off with our elastic net model (See Figure 3 and supplementary notes for a detailed interrogation on the HLA haplotype analysis results). This analysis also suggested which SNPs selected by elastic net that were below conventional threshold could be explained through LD. The second approach was to further reduce the risk of “over-fitting” data with the inclusion of an independent cohort and use of a combined FDR (see methods), which was in addition to the utilisation of cross-validation to set the lambda parameter prior to running elastic net and incorporation of a bootstrap wrapper with subsampling. This enabled the identification of 69 SNPs robustly associated with MS across the three loci (Table 5 and Table S1). A total of 10 SNPs mapped to *TNXB*, including a missense SNP, and is a gene shown in RRMS patients to contain a differentially methylated region identified in CD8+ T cells [68], but not CD4+ T cells [69]. *TNXB* is part of the RCCX module, which includes *CYP21*, *C4* and *STK19*, another hypervariable region (in addition to HLA-DRB) with modular duplication, and a deletion of *C4A* in the presence of *HERV-K* [70,71]. Together, this set of 10 SNPs in *TNXB* may represent genetic variance across the region, with previous studies having also identified linkage across *TNXB*, *CYP21A2* and *AGER* [72] and conflicting reports on the role of *C4* [73,74] in MS. Therefore, these data support further investigations into RCCX allelic and CNV studies in MS.

To garner biological insight into the aetiology of MS, pathway and functional enrichment analysis was performed (Table 5) on the 16 HLA and 12 NKC genes identified (Table 4). In keeping with the involvement of NK cells and innate immunity in MS, pathways involved in bacterial and viral infections were associated as well as MHC class I protein binding. Support for the role of oxidative stress and cellular detoxification were also revealed with the identification of *GPX5*, *GPX6* and *TNF* (see additional files for the full list) from this replication analysis. Oxidative stress has previously been associated with inflammatory conditions such as MS, including altered GPX activity amongst MS subgroups [75], but with little known about the potential underlying genetics causing such associations. Both reactive oxygen species [76,77] and oxidised low-density lipoproteins [78] have been shown to alter NK functionality, which is likely to contribute to disease. This information correlates with the host micro-environment altering NK targets that have been suggested to result in reduced NK functionality in MS patients; since in vitro studies of NK functional assays from MS patients compare to controls has been conflicting [14,79], the evidence now points towards the internal environment of MS patients that dictates NK activity. This coincides with studies that have shown fluctuations of NK function in MS patients [79,80] and reduced oxidative stress (or better control) being associated with a more benign disease course [81].

An exploratory epistasis analysis was performed as the elastic net model was tuned (alpha set toward ridge regression) with the additional aim of capturing interacting SNPs if they are of equal importance to disease status. The results provided suggestive evidence that some of the SNPs revealed in the replication (Table 6) and in-depth discovery cohort analysis (Table S2) were biologically relevant indicating interaction across the HLA, NKC and LRC loci.

This study also highlighted the benefit of considering signals from potentially subtle genetic regional variation (or ‘genetic boundaries’) in concert with individual SNPs that could contribute to disease aetiology. The strength and complexity of signal across the HLA region was evident (Figure 4), with the largest concentration of genetic boundary variance in combination with individual SNPs centred around the HLA-class II, bordering class III (Figure S4). However, HLA-class I also contained some distinctive boundaries with a high signal in combination with a single SNP such as *HLA-F-AS1*. Meanwhile, for the LRC loci, *VSTM1* contained the SNP with the strongest individual SNP signal, whereas *LILRA1* was the gene with the highest boundary signal. In addition, epistasis analysis identified several interactions with a non-coding SNP in *LILRA1* gene with several HLA SNPs (Table S2).

A limitation of the combined analysis was the reduced coverage of SNPs across all three regions due to the use of ‘SNPs in common’ to both cohorts. The benefit of a combined analysis being the identification of a robust set of SNPs associated across two independent cohorts, but at the expense of genetic coverage. Furthermore, the proportion of MS subtypes in the replication cohort was unknown and may not reflect that of the discovery dataset, which could have influenced the results. Therefore, future studies should consider and evaluate potential differences in subtype. Additionally, even the independent discovery cohort analysis was burdened by poor coverage of SNPs in certain regions due to the genotyping platform. For example, the killer immunoglobulin-like receptors (KIRs) loci within the LRC, which has previously been implicated with MS [82,83], was mostly absent from this study even after imputation, which should be addressed in future studies when using these approaches. This limitation is also exacerbated by the fact that elastic net cannot handle missing data, which is why imputation was utilised and good coverage of genetic regions is beneficial prior to imputation.

## 5. Conclusions 

In this study, we have used an elastic net model with stability selection by iterative subsampling to better define three regions of interest in MS aetiology. We identified a robust set of ‘SNP hits’, validated across two independent cohorts and confirmed using summary statistics from a large-scale meta-analysis. The results of this in silico analysis demonstrate the importance of re-analysing GWAS data to reveal biological insight into the MS disease. Future studies could also benefit from incorporating clinical measurements such as disability burden and severity to better understand the biological consequence of genotypes, as well as the consideration of potential differences in MS subtypes. Furthermore, these results provide further evidence for the involvement of NK cells in MS aetiology in a genetic context.

## Figures and Tables

**Table 1:** Cohort summary information for the discovery (ANZgene) and the replication dataset (merged from two IMSGC GWAS and the national blood service as controls. The number of SNPs passing QC is given for each of the three loci, human leukocyte antigen complex (HLA) (green), natural killer cell complex (NKC) (orange) and leukocyte receptor complex (LRC) (blue) from chromosomes 6, 12 and 19, respectively. The number of SNPs is provided for both cohorts, pre-imputation and restricted to common SNPs post- imputation (with a high imputation quality threshold of R^2^ = 0.8). Independent post-imputation analysis was restricted to the discovery cohort (ANZgene) due to the increased number of SNPs available pre-imputation compared to the merged replication dataset, resulting in a much greater yield of high-quality imputed SNPs (R^2^ = 0.8).

		Discovery Dataset (ANZgene)	Replication Dataset (IMSGC + NBS)
Cases	Samples (n)	1617	1313
	F	1172	994
	M	445	319
	F:M	2.6	3.1
Controls	Samples (n)	1988	1458
	F	1231	753
	M	757	705
	F:M	1.6	1.1
Pre-imputed (# of SNPs)	HLA	1047	62
	NKC	137	33
	LRC	122	44
	Total	1306	139
Post-imputation overlapping (# of SNPs)	HLA	2359	
	NKC	2872	
	LRC	520	
	Total	5751	
Post-imputation ANZgene only (# of SNPs)	HLA	54,541	N/A
	NKC	3790	
	LRC	1576	
	Total	59,907	

**Table 2:** Comparison of the five SNPs with the lowest *p* value (2sf) from each loci, using Fisher’s exact testing on the ANZgene dataset. When using Bonferroni correction only SNPs below 5 × 10^−8^ or 4 × 10^−5^ would be considered ‘significant’, representing the gold standard for GWAS or relaxed relative to the number of SNPs analysed, respectively. SNPs, in bold and with an adjacent asterisk, denote those that met the elastic model threshold (Table 3).

	HLA		NKC		LRC	
	rsID	*p* Value	rsID	*p* Value	rid	*p* Value
**SNP**	rs9271366 *	1.83 × 10^−61^	rs11053043 *	9.91 × 10^−4^	rs11672654 *	3.29 × 10^−3^
	rs9267992 *	1.30 × 10^−59^	rs3764021 *	1.08 × 10^−3^	rs13344319 *	0.0136
	rs2395182 *	7.59 × 10^−50^	rs11052552	2.08 × 10^−3^	rs4806741 *	0.0153
	rs3132946	1.83 × 10^−48^	rs10844638 *	2.27 × 10^−3^	rs1671196	0.0269
	rs3129941 *	1.59 × 10^−47^	rs10845080 *	3.57 × 10^−3^	rs10418607 *	0.0270

**Table 3:** List of SNPs above 70% iterations identified by elastic net analysis on the pre-imputed discovery cohort compared to their corresponding *P* value from Fisher’s exact testing. The genetic consequence is given for each SNP identified and intergenic SNP placements represented as INT(gene1_gene2). SNPs identified in bold * were also highlighted in Table 2 as one of the five SNPs with the lowest *p* value for that region. Only four out of the five SNPs for each region (from Table 2) made it above the 70% threshold; the SNPs that did not reach bootstrapping threshold were rs3132946, rs11052552 and rs1671196 for the HLA (green), NKC (orange) and LRC (blue) region, respectively. The shades of colour relates to the elastic net model range of iterations (70–79, 80–89, 90–100).

Chromosome/Loci	rsID	Iterations (%)	*p* Value (Fisher’s Exact Testing)	Gene: Genetic Consequence
6/HLA	rs2395182 *	100	7.59 × 10^−50^	HLA-DRA: 500B Downstream Variant
	rs3117098	100	2.95 × 10^−35^	HCG23: Non Coding Transcript Variant; LOC101929163: Intron Variant
	rs3129941 *	100	1.59 × 10^−47^	C6orf10: Missense Variant; LOC101929163: Intron Variant
	rs6903608	100	3.14 × 10^−32^	INT(HLA-DRB9_HLA-DRB5)
	rs9271366 *	100	1.83 × 10^−61^	INT(HLA-DRB1_HLA-DQA1)
	rs9267992 *	100	1.29 × 10^−59^	INT(NOTCH4_TSPBP1-AS1)
	rs2854050	99.6	7.36 × 10^−10^	NOTCH4: Intron Variant
	rs9277535	99.6	5.45 × 10^−4^	HLA-DPB1: 3 Prime UTR Variant
	rs926070	99.2	2.74 × 10^−36^	TSBP1-AS1: Intron Variant
	rs2281389	98.4	4.73 × 10^−4^	HLA-DPA2: not reported
	rs2394160	98.4	3.00 × 10^−12^	HLA-F: Intron Variant; HLA-F-AS1: Intron Variant
	rs2844482	98.1	0.673	LTA: Intron Variant; LOC100287329: Intron Variant
	rs2647050	96.5	4.00 × 10^−19^	INT(HLA-DQB1_MTC03P1)
	rs2856718	96.5	4.00 × 10^−19^	INT(HLA-DQB1_MTC03P1)
	rs2395150	95.2	3.90 × 10^−29^	C6orf10: Intron Variant; LOC101929163: Intron Variant
	rs1362126	94.3	7.31 × 10^−13^	HLA-F: 2KB Upstream Variant
	rs3130299	94.3	7.53 × 10^−11^	INT(NOTCH4_TSPBP1-AS1)
	rs2301271	94.1	2.35 × 10^−23^	HLA-DQB2: Intron Variant
	rs1611285	93.6	6.58 × 10^−6^	LOC105379663: Non Coding Transcript Variant
	rs7453920	92.9	2.40 × 10^−23^	HLA-DQB2: Intron Variant
	rs2050190	92.7	2.43 × 10^−9^	C6orf10: Intron Variant; LOC101929163: Intron Variant
	rs3819721	92.7	7.70 × 10^−12^	TAP2: Intron Variant
	rs2284178	92.3	3.02 × 10^−15^	HCP5: Non Coding Transcript Variant
	rs2051549	90.2	3.02 × 10^−23^	HLA-DQB2: Intron Variant
	rs1077393	88.9	6.93 × 10^−23^	BAG6: Intron Variant
	rs2647012	88.2	1.10 × 10^−34^	INT(HLA-DQB1_MTC03P1)
	rs9275184	88.1	0.529	INT(HLA-DQB1_MTC03P1)
	rs2395174	87	2.00 × 10^−8^	INT(BTNL2_HLA-DRA)
	rs2394412	86.8	3.35 × 10^−5^	LINC00243: Non Coding Transcript Variant
	rs2894046	86.8	3.35 × 10^−5^	LINC00243: Non Coding Transcript Variant
	rs2975033	86.4	3.48 × 10^−11^	LOC105375010: Intron Variant
	rs9277554	84.9	3.11 × 10^−3^	HLA-DPB1: 3 Prime UTR Variant
	rs2848713	84.8	1.02 × 10^−3^	INT(MICA_LINC01149)
	rs9296057	84.2	3.17 × 10^−4^	LOC100294145: Non Coding Transcript Variant
	rs2517912	84	1.90 × 10^−13^	INT(ZDHHC20P1_HLA-F)
	rs620202	83.4	2.00 × 10^−7^	BRD2: Intron Variant
	rs2395349	82.8	2.37 × 10^−5^	HLA-DPB2: Intron Variant
	rs2260000	82.7	1.25 × 10^−19^	PRRC2A: Intron Variant
	rs6904029	82.6	3.75 × 10^−11^	HCG9: Non Coding Transcript Variant
	rs2071653	82.3	1.10 × 10^−11^	MOG: Intron Variant
	rs3117230	82.2	3.37 × 10^−3^	INT(COL11A2PA1_HLA-DPB2)
	rs719653	81.4	5.24 × 10^−26^	INT(HLA-DQB2_HLA-DOB)
	rs4151657	80.8	9.87 × 10^−19^	CFB: Intron Variant
	rs2064478	79.2	3.69 × 10^−3^	COL11A2PA1: not reported
	rs1035798	78.4	1.24 × 10^−12^	AGER: Intron Variant
	rs3129882	78	3.58 × 10^−23^	HLA-DRA: Intron Variant
	rs2395173	76.9	2.78 × 10^−32^	INT(BTNL2_HLA-DRA)
	rs3135338	76	2.76 × 10^−32^	INT(BTNL2_HLA-DRA)
	rs1611185	75.6	8.21 × 10^−10^	HLA-P: not reported
	rs2299851	74.8	2.32 × 10^−3^	MSH5: Intron Variant; MSH5-SAPCD1: Intron Variant
	rs1737046	74.2	2.77 × 10^−10^	INT(LOC353010_HLA-V)
	rs6941112	72.2	2.51 × 10^−18^	STK19: Intron Variant
	rs12665700	72	0.662	MUC22: Missense Variant
	rs721394	71	0.417	INT(HCG24_COL11A2)
12/NKC	rs10845080 *	93.3	3.57 × 10^−3^	KLRD1: Non Coding Transcript Variant
	rs6488285	91.1	0.0259	LOC101928100: Intron Variant
	rs3764021 *	85.2	1.08 × 10^−03^	CLEC2D: Synonymous Variant
	rs11053043 *	82.7	9.91 × 10^−4^	INT(CD69_KLRF1)
	rs10505741	79.9	0.0179	CLEC2A: Intron Variant
	rs10844780	74.5	0.0103	INT(CD69_KLRF1)
	rs10844638 *	74	2.27 × 10^−3^	INT(CLECL1_CD69)
19/LRC	rs11672654 *	97.3	3.29 × 10^−3^	LOC100421130
	rs6509868	82.5	0.0449	INT(LAIR1_TTYH1)
	rs10411879	82	0.0706	INT(LILRA1_LILRB1)
	rs4806741 *	78.9	0.0153	INT(LILRB2_LILRA5)
	rs11669029	77.6	0.0706	INT(TARM1_OSCAR)
	rs10418607 *	71.5	0.027	INT(LILRA4_LAIR1)
	rs272411	70.9	0.0878	LILRA1: Intron Variant
	rs13344319 *	70.1	0.0136	INT(LAIR1_TTYH1)
	rs2296371	70.1	0.185	LILRP2: Non Coding Transcript Variant

**Table 4:** Summary table for all the identified SNPs with a combined FDR value ≤ 0.1 and their associated genetic regions from the combined analysis. For all SNPs that corresponded to a gene, the total number of SNPs identified and a representative lowest combined FDR value (see appendix for full list) and genetic consequences (with the corresponding number of SNPs for that genetic consequence) is given. For SNPs that mapped to an intergenic region, the closest gene upstream and downstream has been indicated by INT(Gene1_Gene2) with the total number of SNPs and lowest representative combined FDR value. SNPs identified within genes for each loci are highlighted; HLA (green), NKC (orange) and LRC (blue).

CHR/Loci	Gene	Total # of SNPs	Lowest combined FDR Value	Genetic Consequence(s)
**6/HLA**	BRD2	1	0.0767	Intron Variant (1)
	C2	4	3.18 × 10^−^^5^	Intron Variant (4)
	CFB	1	1.38 × 10^−^^3^	Intron Variant (1)
	GPX5	2	6.86 × 10^−^^3^	Intron Variant (1); Non-coding transcript variant (1)
	GPX6	3	6.86 × 10^−^^3^	Intron Variant (2); missense variant (1)
	HLA-DOB	3	0.0133	Intron Variant (1); 2KB Upstream Variant (2)
	KIFC1	1	0.0693	Intron Variant (1)
	LTA	2	0.0129	Downstream Variant (1); Missense Variant (1)
	LOC100287329	2	0.012855556	2KB Upstream Variant (2)
	PHF1	1	0.032153333	Intron Variant (1)
	SYNGAP1	2	0.069256	Intron Variant (2)
	TAP1	1	4.05E-03	Intron Variant (1)
	TAP2	4	1.87 × 10^−^^4^	Intron Variant (3); synonymous Variant (1)
	TNF	1	0.012855556	2K Upstream Variant (1)
	TNXB	10	1.11 × 10^−^^4^	Intron Variant (9); Missense Variant (1)
	WDR46	1	0.0254	Intron Variant (1)
	INT(FKBPL_PPT2)	4	5.56 × 10^−^^7^	NA/Intergenic
	INT(HLA-DOA_HLA-DPA1)	2	0.0118	
	INT(HLA-DQB2_HLA-DOB)	4	4.05 × 10^−^^3^	
	INT(PPP1R2P1_ LOC100294145)	2	4.05 × 10^−^^3^	
	INT(TAP1_PPP1R2P1)	1	1.47 × 10^−^^5^	
	INT(ZBTB9_BAK1)	2	0.0235	
	INT(ZSCAN23_GPX6)	1	0.022343889	
**12/NKC**	CLEC12B	1	0.022577778	Intron Variant (1)
	LOC102724020	1	0.022577778	Intron Variant (1)
	LOC112268091			Intron Variant (1)
	CLEC2A	2	1.74 × 10^−^^3^	Intron Variant (2)
	KLRF2	1	1.74 × 10^−^^3^	Intron Variant (1)
	KLRA1P	2	0.054753333	Intron Variant (1); 2KB Upstream Variant
	KLRB1	1	0.093155556	Intron Variant (1)
	KLRC4-KLRK1 readthrough	1	0.083367778	Intron Variant (1)
	KLRC4	1	0.083367778	2KB Upstream Variant (1)
	LINC02390	1	0.084108444	Non-Coding Transcript Variant (1)
	LOC105369658	1	0.037733333	Intron Variant (1)
	LOC374443, C-type lectin domain family 2 member D pseudogene	2	0.012474	Intron Variant (2)
	INT(KLRB1_CLEC2D)	1	0.023585333	NA/Intergenic
	INT(LINC02446_KLRA1P)	2	0.0377	
	INT(LOC408186_KLRB1)	1	0.0286	
**19/LRC**	RPS9	3	9.78 × 10^−^^4^	Intron Variant (3)
	INT(LILRA2_LILRB1)	2	0.0520	NA/Intergenic

**Table 5:** Top hits from the functional Enrichment analysis by ToppGENE using the identified genes in the overlapping analysis (Table 3) and passing FDR (adjusted *p* < 0.05). The top five biological processes identified all belong to the same lineage of pathways, with leukocyte mediated immunity being a parent pathway of natural killer cell mediated cytotoxicity (numbered 1 to 4). See ssupplementary files ‘additional file S1’ for full results.

GO (Biological Process)	ID	*p*-Value	*q*-Value FDR B & H	Hit Count in Query List	Hit Count in Genome	Hit in Query List
natural killer cell mediated cytotoxicity	GO:0042267	8.59 × 10^−11^	5.45 × 10^−8^	6	72	KLRC4-KLRK1, TAP1, TAP2, KLRF2, CLEC12B, CLEC2A
natural killer cell mediated immunity	GO:0002228	1.11 × 10^−10^	5.45 × 10^−8^	6	75	KLRC4-KLRK1, TAP1, TAP2, KLRF2, CLEC12B, CLEC2A
lymphocyte mediated immunity	GO:0002449	1.18 × 10^−10^	5.45 × 10^−8^	9	407	C2, LTA, TNF, KLRC4-KLRK1, TAP1, TAP2, KLRF2, CLEC12B, CLEC2A
leukocyte mediated cytotoxicity	GO:0001909	3.63 × 10^−9^	1.25 × 10^−6^	6	133	KLRC4-KLRK1, TAP1, TAP2, KLRF2, CLEC12B, CLEC2A
regulation of lymphocyte mediated immunity	GO:0002706	2.54 × 10^−8^	7.03 × 10^−6^	6	184	LTA, TNF, KLRC4-KLRK1, TAP1, TAP2, CLEC12B
regulation of immune effector process	GO:0002697	3.06 × 10^−8^	7.05 × 10^−6^	8	527	C2, LTA, TNF, KLRC4-KLRK1, TAP1, TAP2, CFB, CLEC12B
cell killing	GO:0001906	6.96 × 10^−8^	1.38 × 10^−5^	6	218	KLRC4-KLRK1, TAP1, TAP2, KLRF2, CLEC12B, CLEC2A
GO (Molecular Function)						
tapasin binding	GO:0046980	1.12 × 10^−6^	1.36 × 10^−4^	2	2	TAP1, TAP2
ABC-type peptide transporter activity	GO:0015440	6.68 × 10^−6^	2.04 × 10^−4^	2	4	TAP1, TAP2
ABC-type peptide antigen transporter activity	GO:0015433	6.68 × 10^−6^	2.04 × 10^−4^	2	4	TAP1, TAP2
TAP2 binding	GO:0046979	6.68 × 10^−6^	2.04 × 10^−4^	2	4	TAP1, TAP2
TAP1 binding	GO:0046978	1.11 × 10^−5^	2.65 × 10^−4^	2	5	TAP1, TAP2
carbohydrate binding	GO:0030246	1.31 × 10^−5^	2.65 × 10^−4^	5	295	KLRC4-KLRK1, KLRB1, KLRF2, CLEC12B, CLEC2A
TAP binding	GO:0046977	3.11 × 10^−5^	5.42 × 10^−4^	2	8	TAP1, TAP2
MHC protein binding	GO:0042287	4.15 × 10^−5^	6.34 × 10^−4^	3	63	HLA-DOB, TAP1, TAP2
MHC class Ib protein binding	GO:0023029	8.63 × 10^−5^	1.17 × 10^−3^	2	13	TAP1, TAP2
ABC-type transporter activity	GO:0140359	2.54 × 10^−4^	2.82 × 10^−3^	2	22	TAP1, TAP2
glutathione peroxidase activity	GO:0004602	2.54 × 10^−4^	2.82 × 10^−3^	2	22	GPX5, GPX6
GO (Pathway)						
Antigen processing and presentation	83074 (KEGG)	9.58 × 10^−8^	2.08 × 10^−5^	5	77	TNF, HLA-DOB, TAP1, TAP2, KLRC4
Herpes simplex infection	377873 (KEGG)	7.45 × 10^−6^	8.09 × 10^−4^	5	185	LTA, TNF, HLA-DOB, TAP1, TAP2
Type I diabetes mellitus	83095 (Reactome)	3.74 × 10^−5^	2.41 × 10^−3^	3	43	LTA, TNF, HLA-DOB
Activation of C3 and C5	1269248 (KEGG)	4.73 × 10^−5^	2.41 × 10^−3^	2	7	C2, CFB
Malaria	152665 (KEGG)	5.55 × 10^−5^	2.41 × 10^−3^	3	49	TNF, KLRC4-KLRK1, KLRB1
Staphylococcus aureus infection	172846 (KEGG)	8.30 × 10^−5^	3.00 × 10^−3^	3	56	C2, HLA-DOB, CFB
Antigen Presentation: Folding, assembly and peptide loading of class I MHC	1269194 (Reactome)	6.64 × 10^−4^	2.06 × 10^−2^	2	25	TAP1, TAP2
Regulation of Complement cascade	1269250 (Reactome)	7.76 × 10^−4^	2.10 × 10^−2^	2	27	C2, CFB
Asthma	83120 (KEGG)	1.02 × 10^−3^	2.10 × 10^−2^	2	31	TNF, HLA-DOB
Initial triggering of complement	1269242 (Reactome)	1.02 × 10^−3^	2.10 × 10^−2^	2	31	C2, CFB
Systemic lupus erythematosus	83122 (KEGG)	1.06 × 10^−3^	2.10 × 10^−2^	3	133	C2, TNF, HLA-DOB
Primary immunodeficiency	83125 (KEGG)	1.46 × 10^−3^	2.35 × 10^−2^	2	37	TAP1, TAP2
Detoxification of Reactive Oxygen Species	1270420 (Reactome)	1.54 × 10^−3^	2.35 × 10^−2^	2	38	GPX5, GPX6

**Table 6:** Epistasis results on the panel of SNPs identified by the combined FDR results (Table S2) performed using a merged discovery and replication dataset. Epistatic interactions were only considered for SNPs in different loci for HLA (green), NKC (orange) and LRC (blue), and not within genomic regions.

SNP1		SNP2		Epistasis Interaction	
Chr:bp	Genomic Location	Chr:bp	Genomic Location	OR_INT	P
6:32829320	INT(TAP1_PPP1R2P1)	12:10750157	KLRA1P	0.48	0.000255
6:32832786	INT(TAP1_PPP1R2P1)	12:10750157	KLRA1P	0.487	0.000685
6:32102305	INT(FKBPL_PPT2)	19:55116651	INT(LILRA1_LILRB1)	0.632	0.00306
6:32112626	INT(FKBPL_PPT2)	19:55116651	INT(LILRA1_LILRB1)	0.639	0.00392
6:32069806	TNXB	19:55116651	INT(LILRA1_LILRB1)	0.636	0.00418
6:31916400	CFB	12:9742327	INT(LOC408186_KLRB1)	1.76	0.00526
6:32109165	INT(FKBPL_PPT2)	19:55116651	INT(LILRA1_LILRB1)	0.646	0.00561
6:32026257	TNXB	19:55116651	INT(LILRA1_LILRB1)	0.648	0.00601
6:31879158	C2	19:55116651	INT(LILRA1_LILRB1)	0.662	0.0105
6:31888367	C2	19:55116651	INT(LILRA1_LILRB1)	0.667	0.012
6:31884823	C2	19:55116651	INT(LILRA1_LILRB1)	0.672	0.0137
6:32852448	INT(PPP1R2P1_LOC100294145)	12:9742327	INT(LOC408186_KLRB1)	2.8	0.0162
6:31542308	TNF; LTA; LOC100287329	12:9742327	INT(LOC408186_KLRB1)	0.561	0.018
6:32069806	TNXB	12:10750157	KLRA1P	0.679	0.022
6:32026257	TNXB	12:10750157	KLRA1P	0.682	0.0239
6:32112626	INT(FKBPL_PPT2)	12:10044542	CLEC2A; KLRF2	6.42	0.0287
6:32109165	INT(FKBPL_PPT2)	12:10044542	CLEC2A; KLRF2	6.23	0.0314
6:32938199	BRD2	12:10169041	CLEC12B; LOC102724020; LOC112268091	0.722	0.0423
6:32057972	TNXB	12:10700014	LOC105369658	0.142	0.0432
6:32010272	TNXB	12:10700014	LOC105369658	0.142	0.0434
6:32021838	TNXB	12:10700014	LOC105369658	0.142	0.0434
6:32030284	TNXB	12:10700014	LOC105369658	0.142	0.0435
6:32019746	TNXB	12:10700014	LOC105369658	0.142	0.0436
6:31540556	LTA; LOC100287329	12:9742327	INT(LOC408186_KLRB1)	0.629	0.0467
6:31916400	CFB	19:55116651	INT(LILRA1_LILRB1)	0.785	0.0472

## Data Availability

Discovery dataset: Access to the ANZgene dataset can be obtain upon reasonable request to the data custodian: justin.rubio@florey.edu.au. Replication cohort: Publicly available data from the IMSGC was obtained from the database Genotypes and Phenotypes (dbGaP) (Accession numbers: phs000275; phs000139; phs000171). The national blood donors (NBS) cohort, obtained from the Wellcome Trust Case Control Consortium, was used as controls. Meta-analysis: Request for permission to access to the meta-analysis summary statistic can be made to the Data Access Committee of IMSGC (https://imsgc.net/, accessed on 21 May 2021). Web Resources: USCS table (genome) browser, https://genome.ucsc.edu/cgi-bin/hgTables; Michigan Imputation Server, https://imputationserver.sph.umich.edu; PLINK 1.9, https://www.cog-genomics.org/plink2/; HAPLOVIEW https://www.broadinstitute.org/haploview/haploview; R, https://www.r-project.org/; Genome Reference Consortium (for boundary selection of HLA and LRC) https://www.ncbi.nlm.nih.gov/grc; Kaviar (conversion from chr:bp to rsID), http://db.systemsbiology.net/kaviar/cgi-pub/Kaviar.pl; ToppFun (Functional Enrichment), https://toppgene.cchmc.org/enrichment.jsp; dbsnp, https://www.ncbi.nlm.nih.gov/snp/; Database of Genotypes and Phenotypes (dbGaP), https://www.ncbi.nlm.nih.gov/gap/; European Genome-Phenome Archive, https://ega-archive.org/.

## Supplementary Materials

The following are available online at https://www.mdpi.com/article/10.3390/genes13010087/s1, Supplemental Data: Figure S1: Haplotype structure of the HLA SNPs identified by elastic net with stability selection (Table 2) using haploview; Figure S2: Visual comparison of elastic net results for the discovery and replication cohorts on SNPs in common, post imputation; Figure S3: Visual representation of the distribution (and relative number) of iterations SNPs were identified by elastic net within and across genetic boundaries in the (a) NK and (b) LRC loci; Figure S4: Visual representation of the distribution (and relative number) of iterations SNPs were identified by elastic net within across genetic boundaries in the HLA loci. Table S1: LD analysis by Haploview on the HLA region, focusing on SNPs identified in Table 2; Table S2: SNPs identified by the replication analysis using SNPs in common; Table S3: Epistasis results (*p* < 0.05) using the panel of SNPs ≥ 70% iterations from the elastic net analysis on the independent imputed discovery cohort (Table S3) with an odds ratio (a) above and (b) below 1; Table S4: Comparison of the elastic net model hits from the pre-imputed discovery analysis and combined FDR ‘replication analysis’ to the IMSGC meta-analysis summary statistics (Science chip 2019). Supplementary File S1: Workbook containing (1) Pre-imputed discovery cohort analysis − GLMNet + Fisher’s exact testing; (2) Gene enrichment (GE) analysis results using ToppFun with the genes identified in the replication analysis; (3) Independent discovery cohort boundary analysis results. Supplementary File S2: Gene Boundaries Used. (from the USCS export). See the GitHub page (https://sburnard.github.io/Elastic_Net_MS_GWAS_paper_data/) and the repository (https://github.com/SBurnard/Elastic_Net_MS_GWAS_paper_data) for the interactive html files of the post-imputed imputed discovery analysis (see Figure 4 for the static version and description). Supplementary material and figures will also be made available via this repository.
