# Supplementary material for: Capturing SNP Association across the NK Receptor and HLA Gene Regions in Multiple Sclerosis by Targeted Penalised Regression Models
Source: Genes (Basel). 2021 Dec 29;13(1):87. doi: 10.3390/genes13010087 (PMC8774935; doi:10.3390/genes13010087)
Supplement: Supplementary file 1 [file genes-13-00087-s001.zip › Burnard et al 2022 Supplemental Data.pdf]

# Supplementary Data
# (Figures S1-S4 and supplementary Note)

## Capturing SNP Association across the NK Receptor and HLA Gene Regions in Multiple Sclerosis by Targeted Penalised Regression Models


**Authors:** Sean M. Burnard [1,2], Rodney A. Lea [1,2,3], Miles Benton [4], David Eccles [5], Daniel W. Kennedy [6], Jeannette Lechner-Scott [2,7,8] and Rodney J. Scott [1,9,10]

1. School of Biomedical Sciences and Pharmacy, University of Newcastle, Callaghan NSW 2308, Australia; rodney.a.lea@gmail.com
2. Centre for Brain and Mental Health (CBMHR), Hunter Medical Research Institute (HMRI), New Lambton Heights NSW 2305, Australia; jeannette.lechner-scott@health.nsw.gov.au
3. Centre of Genomics and Personalised Health, School of Biomedical Sciences, Queensland University of Technology, Kelvin Grove QLD 4059, Australia;
4. Human Genomics, Kenepuru Science Centre, Institute of Environmental Science and Research, Wellington 5240, New Zealand; miles.benton84@gmail.com
5. Malaghan Institute of Medical Research, Wellington 6242, New Zealand; bioinformatics@gringene.org
6. Australian Centre of Excellence for Mathematical and Statistical frontiers, Queensland University of technology, Brisbane 4000, Australia
7. School of Medicine and Public Health, University of Newcastle, Callaghan NSW 2308, Australia
8. Department of Neurology, John Hunter Hospital, New Lambton Heights NSW 2305, Australia
9. Division of Molecular Medicine, NSW Health Pathology-North, John Hunter Hospital, New Lambton Heights NSW 2305, Australia
10. Hunter Cancer Research Alliance (HCRA), Hunter Medical Research Institute (HMRI), New Lambton Heights NSW 2305, Australia

# Table of Contents

This supplementary information contains four figures and a more detailed interrogation of the haplotype analysis of the HLA in the discovery dataset. The supplementary tables can be found together in a separate excel workbook.

## Supplementary Figures

**Figure S1.** Haplotype structure of the HLA SNPs identified by elastic net with stability selection (table 2) using haploview……………………………………………………………………………………..1

**Figure S2**. Visual comparison of elastic net results for the discovery and replication cohorts on SNPs in common, post imputation……………………………………………………………………………… 2

**Figure S3.** Visual representation of the distribution (and relative number) of iterations SNPs were identified by elastic net within and across genetic boundaries in the a) NK and b) LRC loci………………………………………………………………………………………….3

**Figure S4.** Visual representation of the distribution (and relative number) of iterations SNPs were identified by elastic net within across genetic boundaries in the HLA loci ………………………………..4

## Supplementary Note

**Detailed interrogation on the HLA haplotype analysis results**…………………………………………5

**a)**

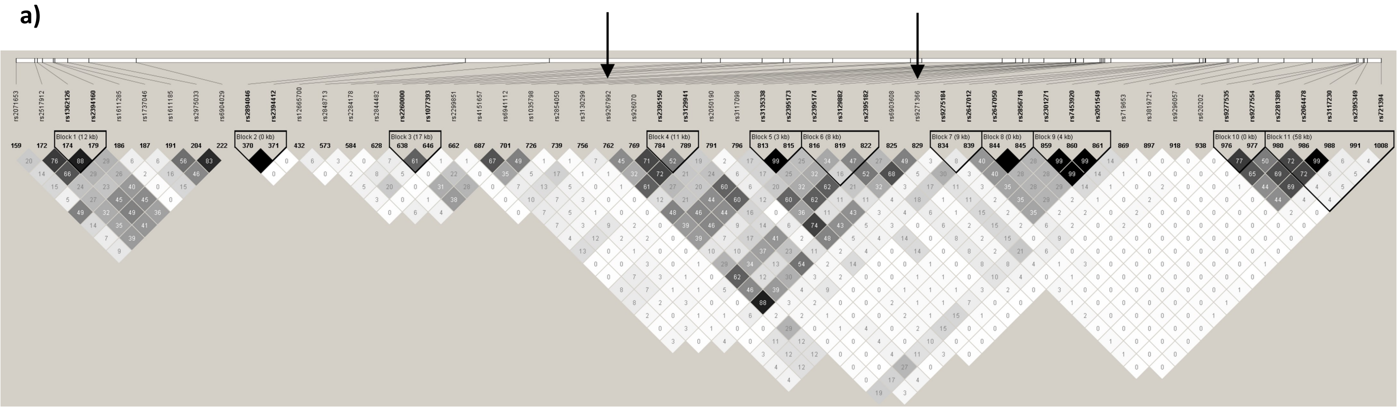

**b)**

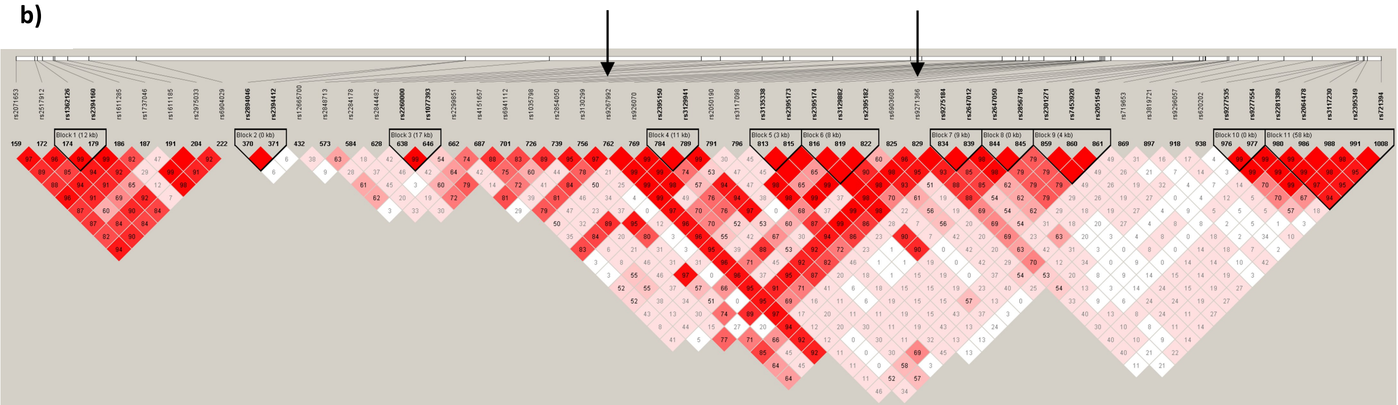

**Figure S1. Haplotype structure of the HLA SNPs identified by elastic net with stability selection (table 2) using haploview**. The rsIDs are given at the top of the display, with the a) $r^2$ or b) D’ values for LD (0-100) between SNPs given in each block running diagonally. As expected, there is a complex underlying LD structure across the HLA region with 11 blocks of inherited SNPs predicted by haploview, consisting of 2-5 SNPs each. Of the 12 SNPs that were identified above 98% iterations from elastic net analysis, only rs9267992 and rs9271366 (black arrows) were determined to be in strong LD and coinherited ($r^2$ >=0.7 and D’ >=0.8). These two SNPs also flank a central set of SNPs (encompassing blocks 4,5 and 6), both with a relatively high level of LD for all SNPs located between them.

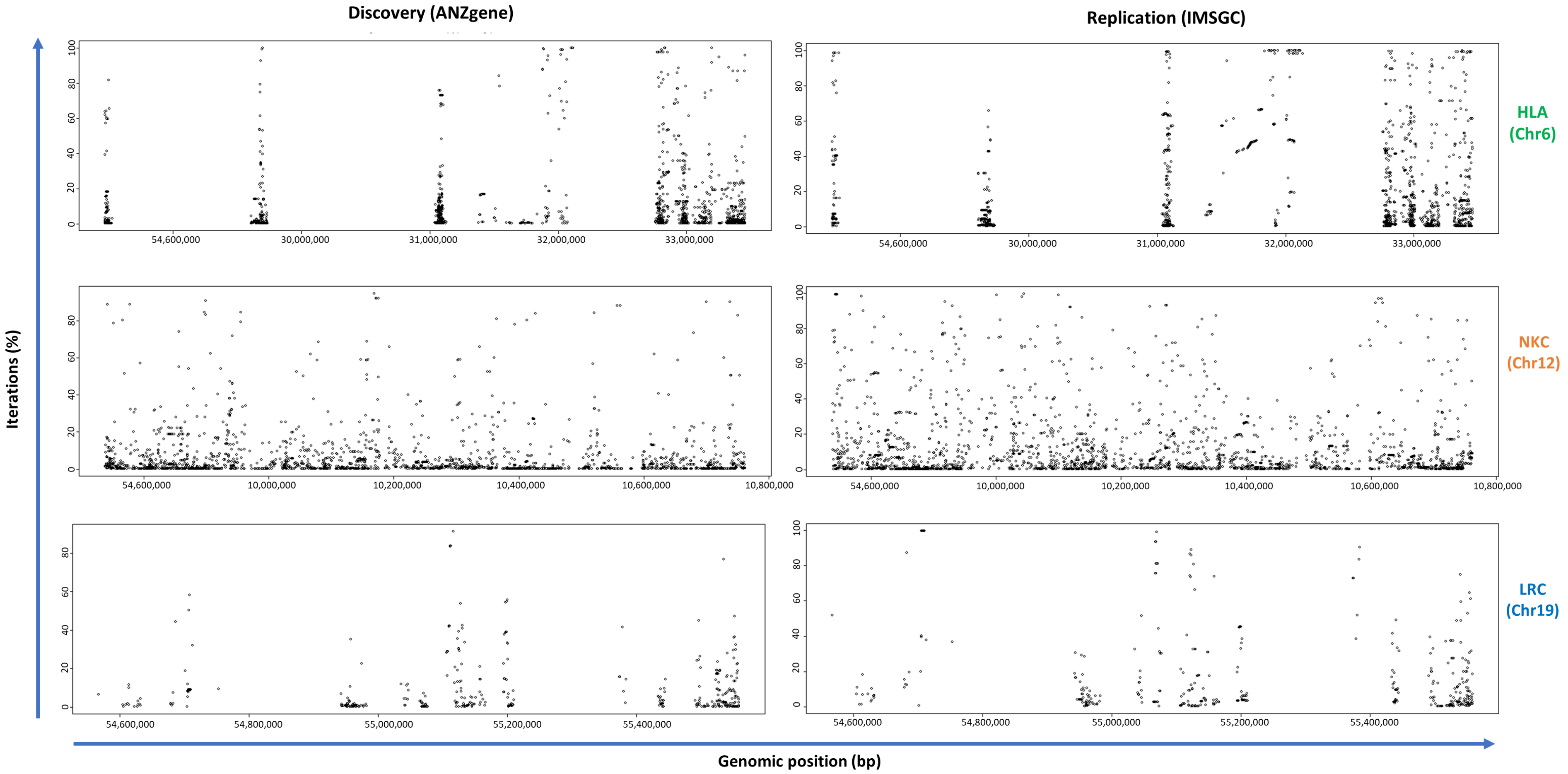


**Figure S2. Visual comparison of elastic net results for the discovery and replication cohorts on SNPs in common, post imputation.** Elastic net model results with the percentage of iterations for each SNP identified are plotted against their genomic position for each region (HLA, NKC and LRC).

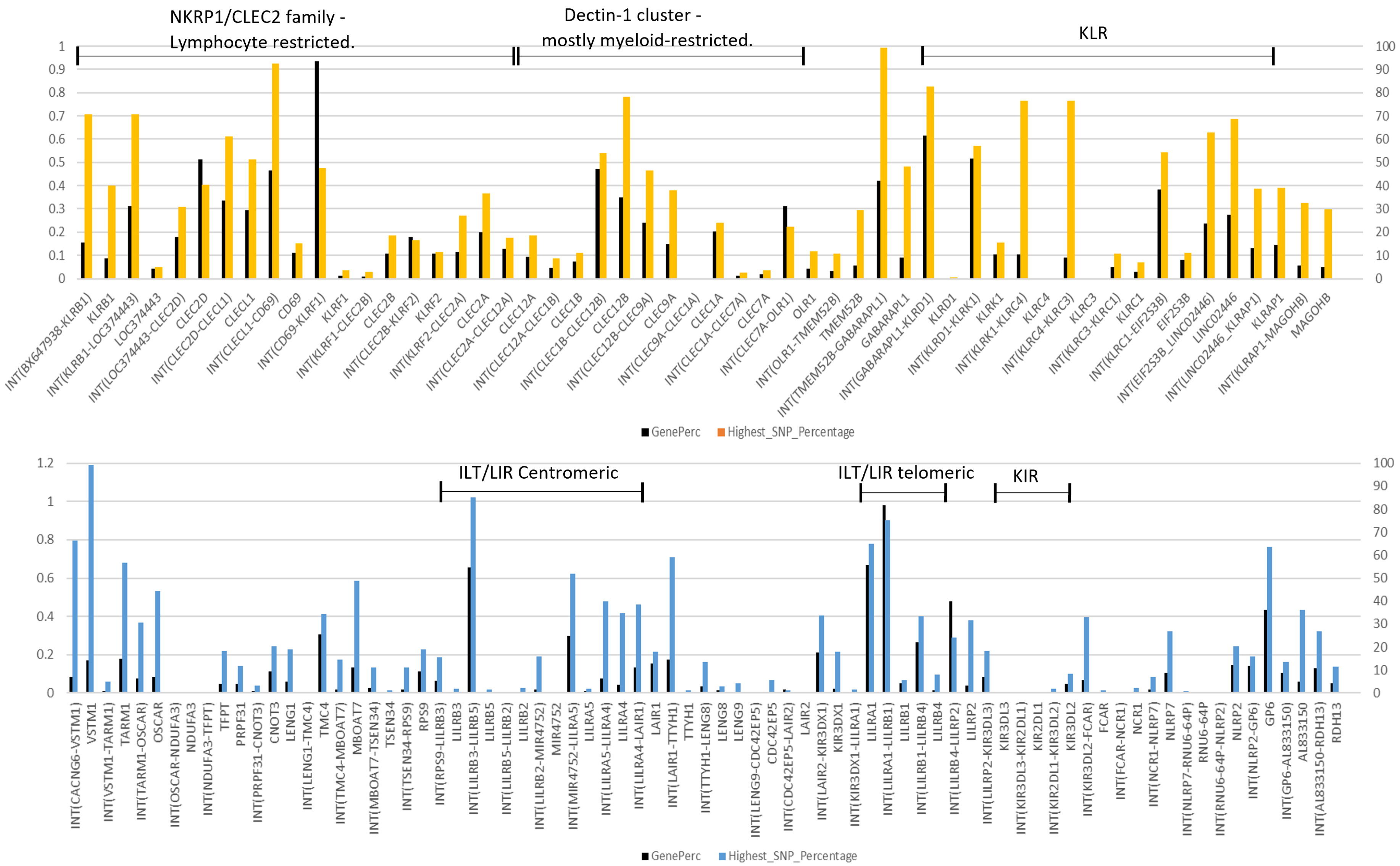


**Figure S3. Visual representation of the distribution (and relative number) of iterations SNPs were identified by elastic net within and across genetic boundaries in the a) NK and b) LRC loci.** Relative number of iterations for each genomic boundary (black bars) as a percentage of total iterations, alongside the representative SNP within each region with the highest percentage of iterations for the **a,** the NKC loci and **b,** the LRC loci represented by orange and blue bars, respectively. Genetic boundaries are in genomic order. The black bars represent the contribution of genetic variation (attributed by many SNPs) within a region to MS while the orange and blue indicate how strong/ consistently an individual SNP within a given region is associated with MS. The general gene clusters have been indicated with bars above A) the NKC and B) the LRC, with an Asterix (*) beside any gene name that belongs to a group outside of the cluster it's physically located.

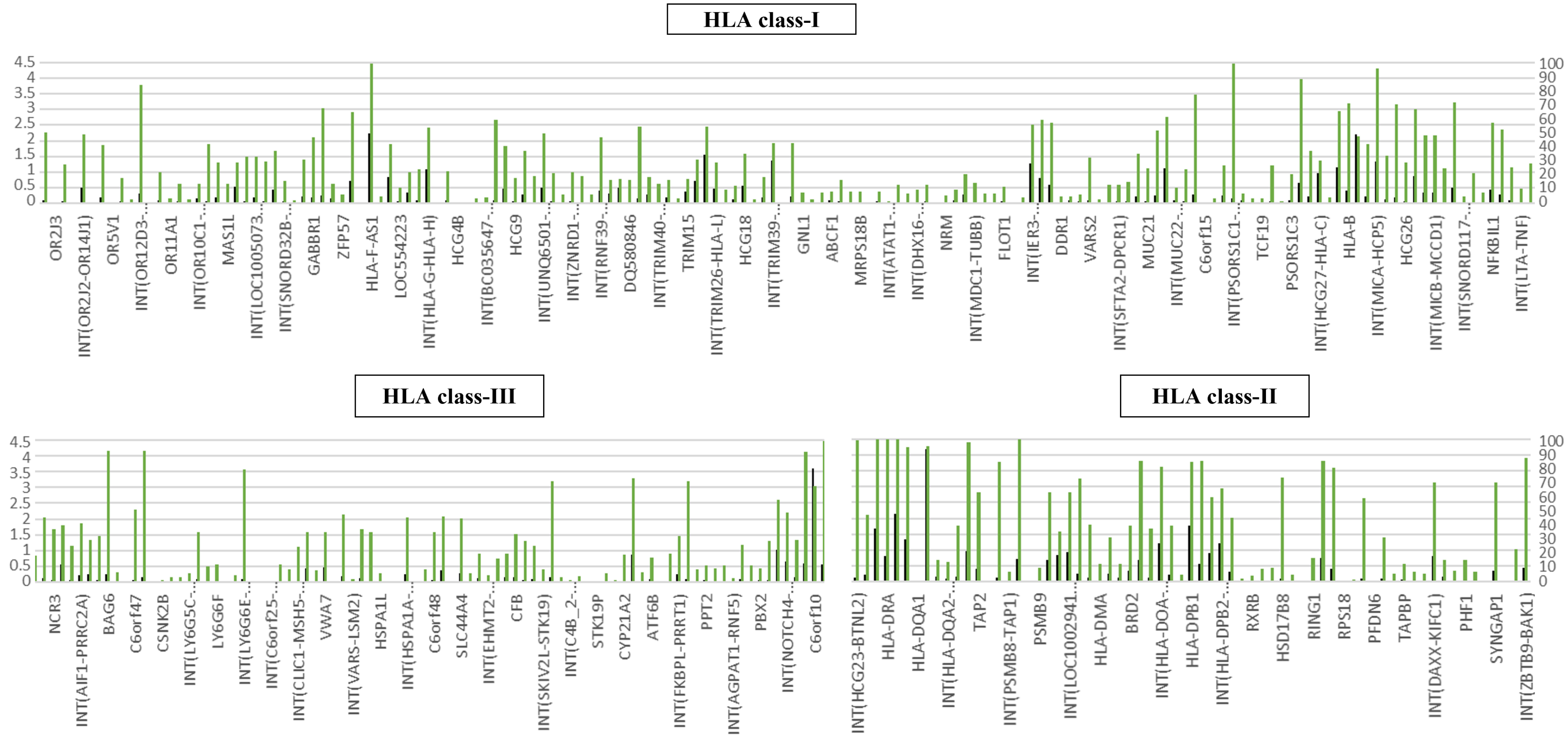


**Figure S4. Visual representation of the distribution (and relative number) of iterations SNPs were identified by elastic net within across genetic boundaries in the HLA loci.** Relative number of iterations for each genomic boundary (black bars) as a percentage of total iterations, alongside the representative SNP within each region with the highest percentage of iterations (green bars). Regions are order in genomic order and separated into HLA class I,III and II. The black bars represent the contribution of genetic variation (attributed by many SNPs) within a region to MS while the green bars indicate how strong/ consistently an individual SNP within a given region is associated with MS.

## Supplementary Note

### Detailed interrogation on the HLA haplotype analysis results

Almost all SNPs showed some level of LD with one or several other SNPs when using D' alone (Figure S1b). When both the $r^2$ and D' values were taken into consideration to identify SNPs that are in strong LD and coinherited (also referred to as a 'proxy SNP'), of the 12 SNPs that reached above 98% iterations, only rs9267992 and rs9271366 (Figure S1, black arrows) met this LD threshold (Table S2). These two SNPs flank a set of SNPs (including the haplotype blocks 4, 5 and 6), showing a varying level of LD for all SNPs between them, which also encompassed all SNPs that reached 100% iterations in the elastic net model (Figure S1). Therefore, this set of SNPs identified from rs9267992 to rs9271366 are likely representative of one central group that may have a profound effect on the risk for MS, which are either coinherited, or at the very least, a mixture of SNPs within this central group are more likely to be observed when either flanking SNP is present. In either case, this risk for MS may indicate alterations in one or several associated genes, beyond the already established HLA-DRB1*1501 risk assigned to rs9271366.

It is also noteworthy that the discrete cluster of the first 9 SNPs at the telomeric end that produced a high D' score (Figure S1b), corresponds to the first peak that reaches above traditional GWAS threshold levels in HLA class I (Figure 1). This first peak in HLA class I culminated in a signal upstream of HLA-F, which is represented by the haplotype block 1 that includes rs2394160 and rs1362126 (and reached 98.4% and 94.3% iterations in our elastic net model, respectively). Therefore, the complex haplotype architecture observed (Figure S1) using the SNPs identified by the elastic net model (Table 2) somewhat mirrors the peaks seen in the Manhattan plot (Figure 2).

The haplotype block 11 included five SNPs ranging from 71.0 to 98.4% iterations spanning across the pseudogenes *HLA-DPA1*, *HLA-DPA2* and *HLA-DPB2*. This signal is toward the centromeric end of *HLA-DPB1*, which is a gene previously established as a risk factor for MS, independent of the *HLA-DRB1*1501* association [1-3], represented by haplotype block 10 (Figure S1 and figure 3). Therefore, the SNPs in haplotype block 11 might represent either an extension of this known risk factor or another independent signal.

### References


1. Hollenbach, J.A. & Oksenberg, J.R. The immunogenetics of multiple sclerosis: A comprehensive review. *Journal of autoimmunity* **64**, 13-25 (2015).
2. Patsopoulos, N.A. *et al.* Fine-mapping the genetic association of the major histocompatibility complex in multiple sclerosis: HLA and non-HLA effects. *PLoS genetics* **9**, e1003926-e1003926 (2013).
3. Field, J. *et al.* A polymorphism in the HLA-DPB1 gene is associated with susceptibility to multiple sclerosis. *PloS one* **5**, e13454-e13454 (2010).
